# Cellular Hallmarks From Volume Electron Microscopy Reveal Developmental Progression of Plasmodium Ookinetes

**DOI:** 10.1002/advs.202508250

**Published:** 2025-09-30

**Authors:** Nedal Darif, Marco Rheinnecker, Kolja Hildenbrand, Thanat Chookajorn, Lilian P. Dorner, Jean‐Karim Hériché, Sara Henriksson, Charlotta Funaya, Franziska Hentzschel, Linda Sandblad, Oliver Billker, Yannick Schwab, Friedrich Frischknecht

**Affiliations:** ^1^ Cell Biology and Biophysics EMBL 69117 Heidelberg Germany; ^2^ Faculty of Biology Heidelberg University 69120 Heidelberg Germany; ^3^ Parasitology Center for Infectious Diseases Heidelberg University Medical Faculty 69120 Heidelberg Germany; ^4^ Laboratory of Molecular Infection Medicine and Department of Molecular Biology Umeå University Umeå 90187 Sweden; ^5^ Umeå University Faculty of Science and Technology Department of Chemistry. SciLifeLab research infrastructure at Umeå University Umeå Centre for Electron Microscopy Umeå 90736 Sweden; ^6^ Electron Microscopy Core Facility Heidelberg University 69120 Heidelberg Germany; ^7^ German Center for Infection Research DZIF partner site 69120 Heidelberg Germany; ^8^ Collaboration for joint PhD degree between EMBL and Heidelberg University Faculty of Biosciences 69117 Heidelberg Germany

**Keywords:** developmental biology, malaria, Plasmodium, single cell development, ultrastructural atlas, volume electron microscopy

## Abstract

Unicellular organisms or cells of metazoans often change their morphology during development or life cycle progression to adapt to environmental changes. Malaria parasites undergo a striking range of morphological transformations as they navigate through the different environments of mammalian hosts and mosquito vectors. These developmental transitions are accompanied by changes in the subcellular organelles. Here, this work introduces an unbiased approach using volume electron microscopy (vEM) to facilitate cluster analyses of morphometric parameters during developmental transformation. Investigating the transformation of fertilized *Plasmodium* zygotes into the motile ookinetes with three complementary vEM techniques revealed intimate mitochondrion‐nucleus interactions, different microtubule arrangements, elongated shapes of micronemes and their close interaction with the apicoplast. The presented data and approach provide an open‐access subcellular atlas for ookinete development to aid mechanistic molecular insights from reverse genetic studies and a framework for the ultrastructural study of other parasite stages and developmental transitions in general.

## Graphical abstract

This article presents a detailed ultrastructural cell atlas of *Plasmodium* ookinete development using advanced volume electron microscopy (v) techniques. It defines seven distinct developmental stages, revealing complex organelle interactions and offering new insights into parasite differentiation. The work highlights the usage of morphometric parameters obtained from vEM for cell stage determination.

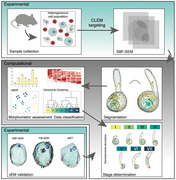

## 1 Introduction

**Figure 1:**
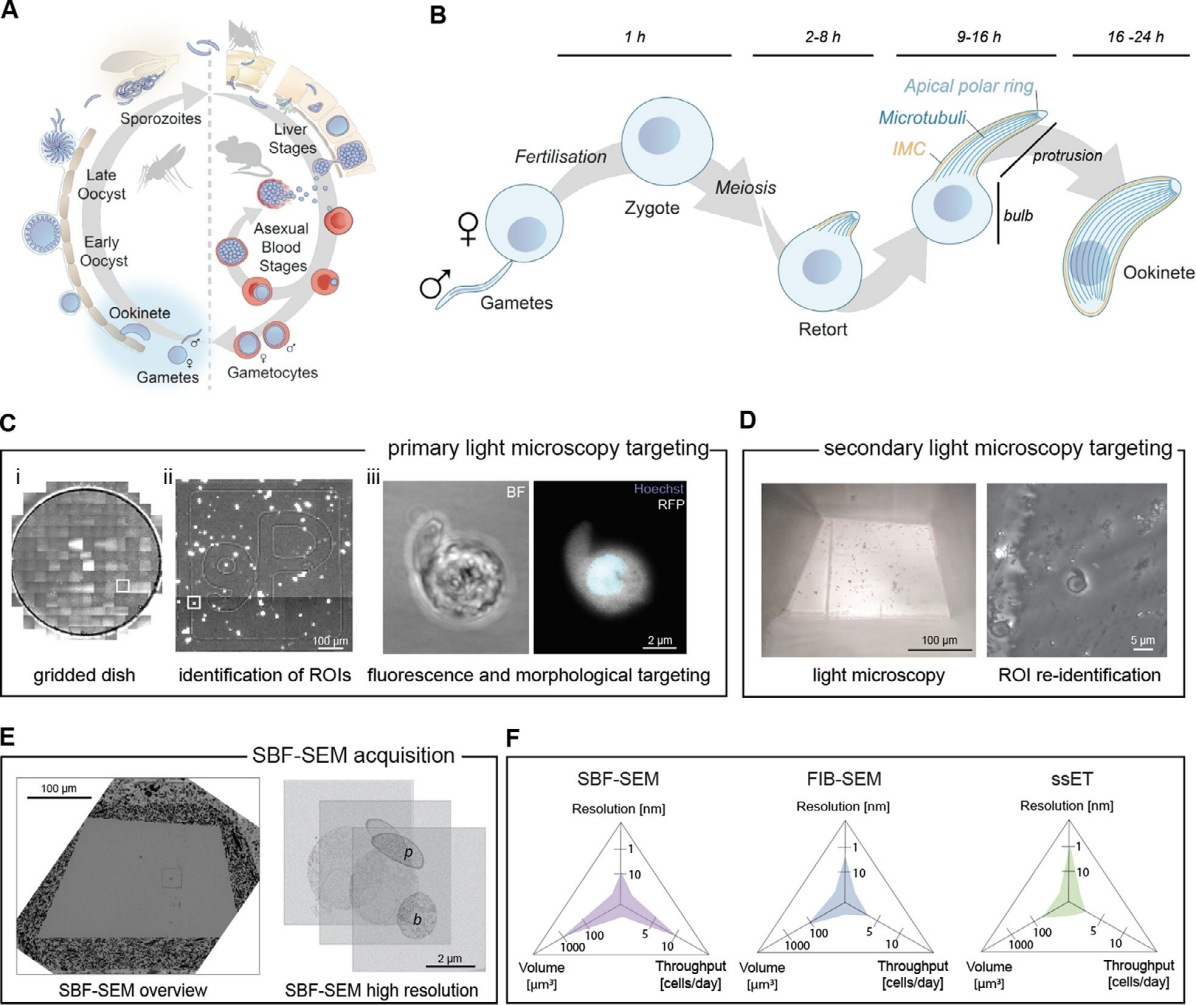
Overview of biological framework and methodological concepts. A) Life cycle of rodent infecting *Plasmodium berghei*, light blue: stages relevant for this study as detailed in B. B) Schematic representation of ookinete development from the fusion of male and female gametes to development of the zygote, the retort and the final ookinete. The retort features two major parts, a growing cellular protrusion and a shrinking spherical bulb. Approximate timing for the different stages as they apply to *P. berghei*. C) Primary light microscopy targeting of cells on MatTek dishes of interest. i: overview of whole gridded dish containing ≈10 000 cells in ≈100 quadrants (white box). ii: Single quadrant containing ≈100 cells. iii: single retort targeted for SBF‐SEM. D) Secondary light microscopy targeting on the SBF‐SEM block after electron microscopy sample preparation identifies the same cell as in C_iii_. E) SBF‐SEM view of targeted block in overview and three stacked images from high resolution acquisition revealing the bulb (b) and protrusion (p) of the retort from C_iii_. F) Three major characteristics of different volume EM techniques utilized in this study, highlighting the advantages and challenges of each technique.

Developmental transitions to generate different cell types are essential for both multicellular and unicellular organisms. While development in multicellular organisms is mostly unidirectional from stem cells to terminally differentiated cells, development in unicellular organisms can occur in complex cyclical manners as most impressively exhibited by parasitic protozoa. Among those, parasites of the genus *Plasmodium* cause malaria and are the most medically significant, as they continue to impose a heavy burden on endemic countries. Malaria parasites undergo a cyclical development between mosquito vectors and mammalian hosts, altering their molecular and organellar constitution to adapt to each environment and its specific demands. These changes have been detailed with transcriptional analysis as well as with 2D electron microscopy (EM) and in a few cases also 3D EM.^[^ ^1^, ^2^, ^3^, ^4^, ^5^, ^6^ ^]^ Advancements in understanding malaria biology have consistently paralleled technological progress in imaging.^[^ ^7^ ^]^ The introduction of 3D or vEM techniques allows deeper insights into the ultrastructural changes of organelles and cytoskeletal structures.^[^ ^8^ ^]^ Similar to the analysis of, for example, scRNAseq experiments, vEM also offers the opportunity for an unbiased cluster analysis of morphological parameters of the imaged data using dimensionality reduction such as principal component analysis.^[^ ^9^, ^10^, ^11^ ^]^ This has already allowed the identification of distinct cellular groups within a single organism solely from imaging data, but has not yet been applied for the study of developmental transitions.^[^ ^10^ ^]^ Here, we explored the use of vEM to study the morphogenesis of *Plasmodium* ookinetes, the motile parasites generated from zygotes in the mosquito midgut just hours after the insect took an infectious blood meal (**Figure** **1**A). We deemed these ideal model cells to study developmental transitions due to their discernible developmental progression and ease of culture.

Upon ingestion by the mosquito host, the female and male *Plasmodium* gametocytes differentiate into gametes, which in turn fuse to form a diploid zygote. Subsequently, the round zygote develops into an elongated and motile ookinete, which invades the midgut epithelium and transforms into an oocyst, where sporozoites are generated to ultimately transmit back to the mammalian host. Ookinete morphological development begins with the formation of a protrusion that establishes the apical end of the highly polarized cell. At the apical end a polar ring is formed from where microtubules are polymerized, driving the elongation of the cell, now called a retort (Figure 1B). Ookinetes were first observed in 1898 in bird malaria parasites and later explored using electron microscopy (Reviewed in Singer and Frischknecht^[^ ^12^ ^]^).^[^ ^13^ ^]^ With the establishment of ookinete cultures and genetic manipulation in the rodent malaria parasite *P. berghei*, detailed examinations of subcellular ookinete compartments and molecular analyses of their development and function were possible.^[^ ^12^, ^14^, ^15^, ^16^ ^]^

To date, external morphology has been a valuable criterion for staging ookinete development, spanning the initial formation of the apical protrusion from the zygote that elongates and eventually gives rise to the crescent‐shaped ookinete. Intermediate stages between the zygote and mature ookinete have been described essentially based on either the length of the protrusion or the time post‐fertilization.^[^ ^17^, ^18^, ^19^, ^20^, ^21^ ^]^ Yet, little information is available about the subcellular processes underlying these transitions.

Here, using light microscopy to identify cells of interest followed by serial block‐face scanning electron microscopy allowed the imaging of 28 cells at different developmental stages and subsequent extraction of morphological parameters. Their unbiased clustering allowed us to define the (sub)cellular hallmarks of zygote‐to‐ookinete transition, which offer new clarity on stage definitions. Together with data from focused ion beam scanning electron microscopy (FIB‐SEM) and serial section electron tomography (ssET) which provide higher resolution and thus allow, for example, imaging of individual microtubules, they also provide an ultrastructural atlas to guide functional studies through genetic modifications.

## 2 Results

### 2.1 Establishing a Light Microscopy Targeting Method for SBF‐SEM of Ookinetes

A major limiting factor for vEM is throughput, where acquiring a single cell volume proves to be a methodological and financial challenge. To overcome some of these limitations, the precise targeting of cells of interest can increase throughput and reduce the amount of unintended data acquired.^[^ ^22^, ^23^ ^]^ We thus adopted a light microscopy targeting method that permits the acquisition of several *P. berghei* ookinetes from in vitro culture by serial block‐face scanning electron microscopy (SBF‐SEM) (Figure 1C–E). To visualize ookinetes, we used a parasite line expressing cytoplasmic RFP.^[^ ^24^ ^]^ Standard protocols for in vitro development and density‐gradient enrichment of ookinetes, result in a cell population containing a mix of zygotes and ookinetes at different developmental stages, as well as a range of other cells, including murine leukocytes and erythrocytes infected by asexual blood stage parasites. For light microscopy targeting, we purified and activated gametocytes, induced ookinete development and incubated them for 8, 16, or 20 h. The resulting cell populations were added to a gridded dish with coordinates for correlative experiments. Next, the cells were imaged with a wide‐field light microscope and regions of interests (ROIs) containing several cells of interest were selected based on the morphology, DNA staining and cytoplasmic RFP signal of the cells (Figure 1C, primary light microcopy targeting). Subsequently, the sample was prepared using classic chemical fixation protocols for EM and the resulting blocks were re‐imaged confirming the presence of the cells in the ROI at the corresponding coordinates (Figure 1D, secondary light microscopy targeting). The trimmed block was transferred to the SBF‐SEM, where a block overview to identify the ROIs and subsequently high‐resolution images of the ookinetes were acquired (Figure 1E). This correlative light and electron microscopy workflow (detailed in the Method section) enabled us to efficiently target and image 28 cells by SBF‐SEM. These were selected for their morphology recapitulating different developmental stages, ensuring that at least duplicates of each potential stage are covered. Of these only three cells could not be used for cluster analyses as they either did not yield a full volume (two cells) or lacked a clearly visible nucleolus (one cell). Organelles in each cell were identified visually and segmented using Amira software. Visual inspection of these segmented organelles yielded a large dataset of distinct features, some of which were clearly appearing at distinct developmental stages, such as the presence of nuclear protrusions (named tails and appendices) during early development followed by the presence of micronemes at later stages. We also prepared ookinetes for serial section tomography (ssET) and FIB‐SEM analysis, which offer higher lateral and axial resolution (Figure 1F) and the opportunity for isotropic imaging, always using classical fixation protocols to be able to compare our datasets within this study and published work. In the following we first provide the cluster analysis of the different features that allow staging before detailing the new insights into the individual subcellular features.

### 2.2 Clustering of Ookinetes Based on Distinct Cell Morphologies and Organellar Characteristics

**Figure 2:**
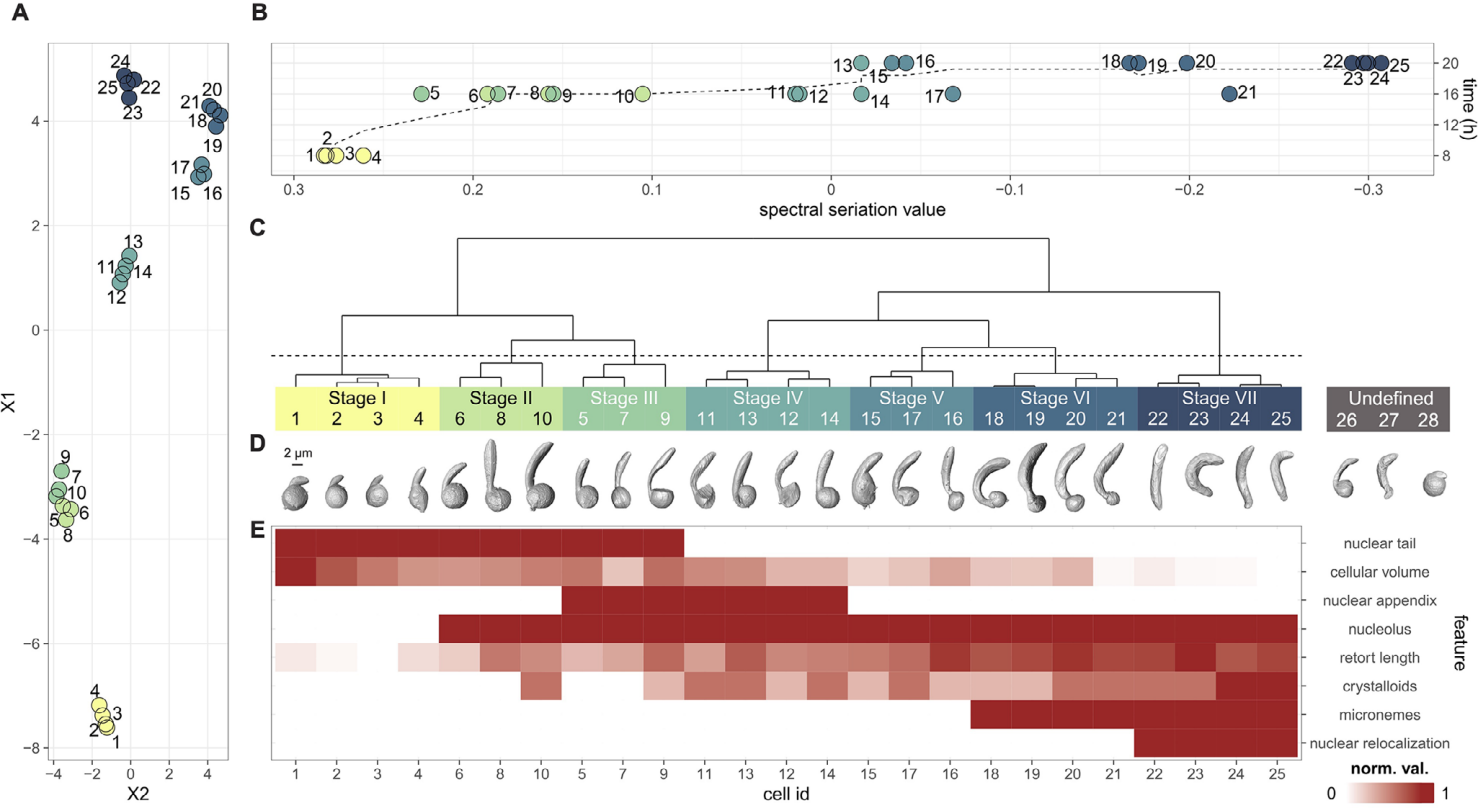
Clustering analysis and key cellular features of retort to ookinete transition. For all different cluster methods the features in E were used, all acquired features can be seen in Figure S1D, Supporting Information. A) UMAP analysis of all cells acquired with SBF‐SEM using the morphometric parameters (features) listed in panel E. B) Spectral seriation analysis of all cells acquired with SBF‐SEM of the three sampling points at 8, 16, and 20 h post gametocyte activation. The different data points in the seriation are plotted against time post gametocyte activation. Fitted dashed line indicates interpolation between ranked data points. C) Dendrogramm from hierarchical clustering analysis from SBF‐SEM data allows subclustering of several stages. D) Whole cell surface rendering of all cells analyzed in the cluster analysis and arranged according to results from C. E) Heatmap of intensity of features used for seriation and cluster analysis. The features are scaled values from 0 to 1, with the detailed scaling described in the Experimental Section, features, full volumes from segmentations, or number of present organelles.

Investigating the data, we noticed that some sub‐cellular features were not present at all stages and decided to compile all of them to perform an unbiased data‐driven approach for classifying and characterizing the cellular development of ookinetes. We therefore selected and quantified from the segmented SBF‐SEM datasets a large set of features including cellular volume, length of retort protrusion, nuclear location, the number of crystalloid bodies and the presence of a nuclear appendix, a nucleolus, or micronemes (**Table** **1**, Figure S1, Supporting Information). This allowed us to generate a correlative coefficient matrix (Figure S1A, Supporting Information) that showed the utility of the eight selected features for subsequent cluster analyses (presented in bold in Figure S1A, Supporting Information). To ensure solid classifications of the different developmental stages, we used dimensionality reduction analyses including UMAP (**Figure** **2**A), PCA (Figure S1B,C), spectral seriation (Figure 2B) and hierarchical clustering (Figure 2C). Hierarchical clustering revealed five main developmental stage clusters, two of which have the potential for further subdivision. Using spectral seriation, we demonstrated through the correlation of the assigned ranks and the original fixation time points, that the selected features are suitable for the pseudo‐temporal arrangement of developmental stages (Figure 2B).^[^ ^25^ ^]^ Ultimately, we defined seven clusters to achieve optimal data representation (Figure 2C). Full cell 3D segmentations (Figure 2D) were aligned under the dendrogram associated with their corresponding stage identifications, I‐VII. A heatmap with the intensity of the analyzed characteristics is shown in Figure 2E by considering the seven stages from the hierarchical clustering, the correlative coefficient matrix, and the results of the PCA. A heatmap of all the features considered in this analysis can be found in Figure S1D, Supporting Information. The heatmap treats cellular volume and retort protrusion length as continuous variables. The crystalloids were counted as discrete numbers and are displayed accordingly in Figure 2E. The remaining features, nuclear tail, nuclear appendix, nucleolus, micronemes, and nuclear relocalization, were treated as binary (present or absent). These data show that we can robustly distinguish seven stages of ookinete development based on morphometric features.

**Table 1:** Each cell is numbered according to the seriation ID (compare Figure 2D). For volumes and surface area the units are given in the row titles. Presence of Micronemes, nuclear tails and nuclear appendices or if the nucleus has been relocalized in the cell has been numerated with 0 (not present/not relocalized) or 1 (present/relocalized), respectively. Absolute numbers for crystalloids and the apicoplast branches were noted. id: seriation identification number; cel. vol.: cellular volume; cel. area: cellular area; cel. sph.: cellular sphericity; nuc. vol.: nuclear volume; nuc. area: nuclear area; nuc. sph.: nuclear sphericity; nuc. tails: nuclear tails; nuc. app.: nuclear appendix; nuc. reloc.: nuclear relocalization; s.c.: synaptonemal complexes; nucl. vol.: nucleolar volume; mn: micronemes; crys: crystalloids; api. vol.: apicoplast volume; api. area: apicoplast area; api. br.: apicoplast branching.

id	cel. vol. [µm^3^]	cel. area [µm^2^]	cel. sph.	retort length [µm]	nuc. vol. [µm^3^]	nuc. area [µm^2^]	nuc. sph	nuc. tail	nuc. app.	nuc. rel.	s. c. [µm^3^]	nucl. vol. [µm^3^]	mn	crys	api. vol.[µm^3^]	api area [µm^2^]	api. br.
1	93,10	117,08	0,56	4,03	12,00	29,70	0,85	1	0	0	0,18	0,00	0	0	0,17	3,01	1
2	82,72	115,30	0,80	3,27	11,03	27,70	0,87	1	0	0	0,16	0,00	0	0	0,15	2,77	4
3	74,05	109,57	0,86	2,72	9,52	25,50	0,85	1	0	0	0,22	0,00	0	0	0,14	2,82	3
4	67,65	100,25	0,80	4,81	8,62	22,30	0,91	1	0	0	0,13	0,00	0	0	0,13	2,95	2
5	74,75	131,71	0,76	7,01	8,98	23,36	0,83	1	1	0	0,22	0,02	0	0	0,11	1,70	3
6	67,11	149,18	0,54	5,71	8,40	22,20	0,90	1	0	0	0,04	0,01	0	0	0,07	1,69	3
7	55,85	101,05	0,70	8,12	6,45	15,12	0,89	1	1	0	0,07	0,03	0	0	0,11	2,08	2
8	69,21	159,68	0,59	10,97	8,37	21,04	0,88	1	0	0	0,09	0,03	0	0	0,11	1,89	1
9	76,80	137,81	0,56	11,98	8,93	18,72	0,89	1	1	0	0,00	0,02	0	1	0,13	1,91	1
10	72,97	142,11	0,69	9,83	9,75	21,68	0,95	1	0	0	0,07	0,02	0	2	0,10	1,93	4
11	70,20	139,70	0,38	8,53	8,37	22,88	0,81	0	1	0	0,09	0,02	0	2	0,18	2,89	3
12	59,74	121,26	0,61	10,12	7,85	22,32	0,79	0	1	0	0,00	0,02	0	1	0,08	2,06	4
13	69,39	134,49	0,61	12,76	7,89	25,70	0,75	0	1	0	0,01	0,03	0	2	0,07	1,63	4
14	59,59	126,55	0,58	10,45	8,96	18,96	0,88	0	1	0	0,00	0,03	0	2	0,10	1,89	5
15	52,28	107,56	0,63	10,74	6,74	19,50	0,89	0	0	0	0,00	0,02	0	1	0,09	1,87	3
16	63,50	116,35	0,66	14,85	6,32	18,40	0,90	0	0	0	0,00	0,02	0	1	0,10	2,10	4
17	55,85	131,00	0,63	11,75	6,52	16,80	0,93	0	0	0	0,00	0,02	0	2	0,14	2,83	1
18	55,37	127,73	0,55	13,24	6,57	25,90	0,76	0	0	0	0,00	0,02	1	1	0,09	2,22	3
19	55,11	118,49	0,58	13,90	6,26	18,60	0,89	0	0	0	0,00	0,02	1	1	0,13	2,82	3
20	59,29	131,71	0,56	15,05	6,22	19,20	0,85	0	0	0	0,00	0,02	1	2	0,11	2,23	3
21	43,32	114,71	0,67	14,01	5,11	15,12	0,88	0	0	0	0,00	0,02	1	2	0,10	1,86	3
22	45,81	103,77	0,60	14,10	4,20	14,80	0,85	0	0	1	0,00	0,02	1	2	0,12	3,06	3
23	43,38	96,09	0,62	15,69	3,66	12,70	0,90	0	0	1	0,00	0,02	1	2	0,09	1,96	3
24	43,03	96,95	0,61	13,11	3,67	12,80	0,90	0	0	1	0,00	0,02	1	3	0,10	2,65	3
25	41,61	92,11	0,63	14,14	4,46	14,60	0,90	0	0	1	0,00	0,01	1	3	0,06	1,75	4
26	NA	NA	0,66	NA	7,17	20,39	0,88	0	0	0	0,00	0,03	0	2	0,10	2,79	1
27	34,61	85,15	0,60	12,71	3,44	13,30	0,83	0	0	1	0,00	NA	1	2	0,03	1,05	3
28	NA	NA	0,93	2,55	12,15	29,70	0,86	1	0	0	0,16	0,00	0	0	0,11	2,34	3

### 2.3 Cellular Morphological Changes During Ookinete Development

**Figure 3:**
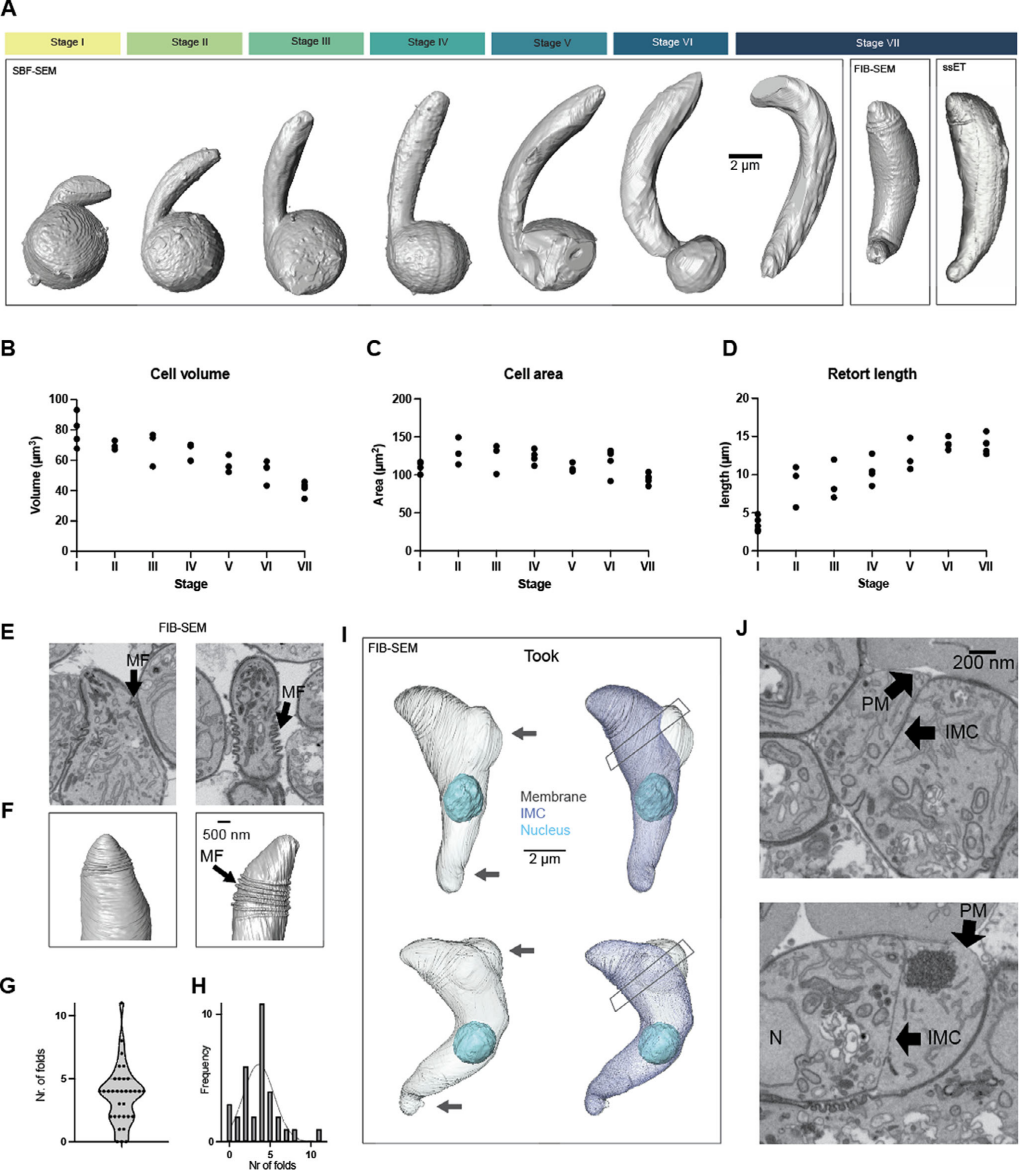
Whole cell segmentation and analysis of retorts and ookinetes using SBF‐ and FIB‐SEM. A) Whole cell representations for one cell of each stage in SBF‐SEM as well as Stage VII for FIB‐SEM and ssET. B–D) Measured cell volumes (B), areas (C) and retort protrusion length (D) from SBF‐SEM data. E–H) Analysis of ookinete pellicular folds. E) FIB‐SEM single slices with arrows pointing at pellicular membrane folds (MF). F) Respective FIB‐SEM volume segmentations from ookinetes in E. G,H) Number and frequency of pellicular folds. I) Volume segmentations of ookinetes transforming into early oocysts (took: transition stage from ookinete to oocyst), showing plasma membrane, IMC and nucleus; grey arrow indicates regions, where the IMC opens up and the plasma membrane bulges out, as described for early oocyst formation (Carter et al., 2007), box indicated position of single slices in J. J) Single slices of the openings at the neck, showing intact organellar compartments, PM: plasma membrane; IMC: inner membrane complex.

To characterize the general cellular attributes of the cells, we generated representative full‐cell 3D segmentations of the plasma membrane from the SBF‐SEM data over the course of ookinete development and quantified specific morphological features such as size and shape. Representative segmentations of cells from each proposed stage showcase the external morphological changes that occur during ookinete development (**Figure** **3**A), from early retort to mature ookinete. To assess if cellular volume and surface area change over the course of development, we quantified these. This revealed that the cell volume decreased from an average of 80 µm^3^ to ≈40 µm^3^ by stage VII (Figure 3B). From stage I to stage VI, the cell surface area stayed between 100 µm^2^ and 150 µm^2^ and decreased just below 100 µm^2^ at stage VII (Figure 3C), while at the same time, the length of the parasite increased from stage I, at 3 µm, to 13 µm with the mature ookinete (Figure 3D). This dramatic reduction in volume suggests the presence of osmotic regulation during ookinete development.

Next, we aimed to analyze ookinete ultrastructure in more detail. This necessitated the use of imaging techniques that provide higher resolution. We therefore employed two other electron microscopy imaging modalities, FIB‐SEM and ssET, to investigate ookinetes that were fixed 24 h after gametocyte activation. This allowed us to image the apical section of additional 33 ookinetes as well as 12 ookinetes across their full volume by FIB‐SEM. Of those only one ookinete was not fully developed, potentially indicating stalled development of a parasite. For ssET we generated 23 serial sections and recorded 57 tomograms to reconstruct a single full 3D ookinete.

FIB‐SEM acquisition enabled us to reliably identify pellicular folds at the apical end of ookinetes (Figure 3E), which were previously reported in single sections or SEM.^[^ ^20^, ^26^ ^]^ These folds comprise both the plasma membrane and the subtending IMC and can appear as intercalated spirals in ookinetes showing a large number of them (Figure 3E,F). 90% (30/33) of the analyzed ookinetes showed at least one membrane fold, with a mean of 3.6 folds per cell and a mean fold‐to‐fold distance of ≈100 nm (Figure 3G,H). These membrane folds may represent an imbalance of membrane reorganization at late ookinete stages possibly as the parasite secretes micronemes during migration.

Next, we assessed the changes in cytoskeletal structures. FIB‐SEM analysis in the resolution and imaging mode we used did not reliably show microtubules. We therefore used ssET, which readily revealed microtubules. ssET showed that the subpellicular microtubules are aligned to the IMC but detached from areas where the IMC is folded (Figure S2). The FIB‐SEM datasets also showed several ookinetes in the early transition phase to an oocyst (termed took), which is characterized by the detachment of the IMC from the plasma membrane.^[^ ^27^ ^]^ At these positions, the membrane bulges out and the cell extends (Figure 3I, grey arrows), ultimately permitting the transfer of organelles to these compartments (Figure 3J). This indicates a flexible attachment of microtubules to the IMC.

### 2.4 Nuclear Morphology is Highly Dynamic

**Figure 4:**
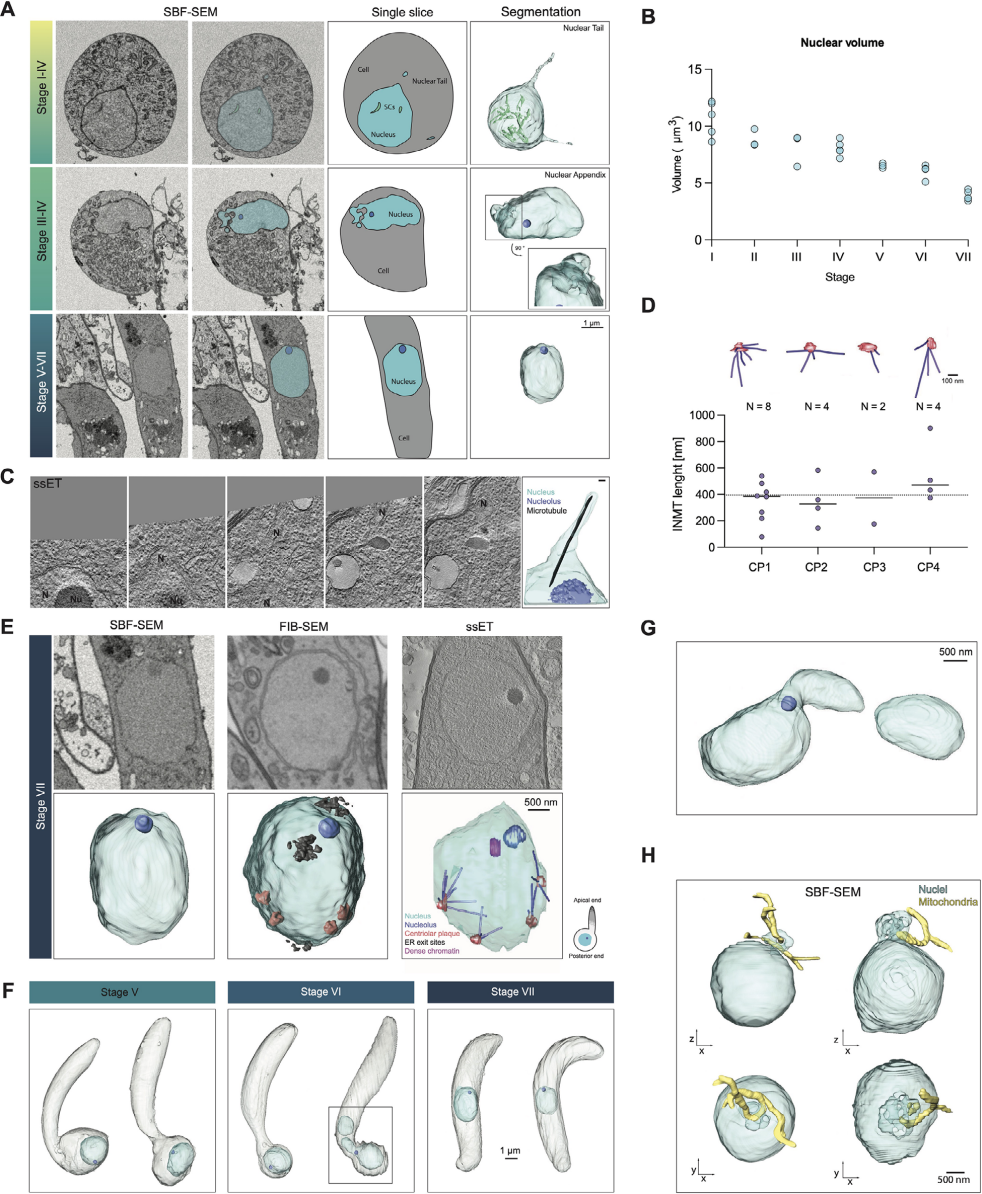
Nuclear features and nucleus‐mitochondrion interactions during ookinete development. A) Three major nuclear features occurring during ookinete development and distributed over stage I to VII from SBF‐SEM data. B) Nuclear volume across the different stages of ookinete development. Three to four nuclei were quantified per stage. C) Nuclear tail of a developing ookinete, containing a single microtubule as revealed by ssET, scale bar 50 µm. D) Centriolar plaques of attached microtubules from ssET and length measurements. E) Stage VII nuclei from all used vEM techniques, showcasing different levels of obtainable detail. F) Visualization of nuclear relocalisation from Stage V to Stage VII from SBF‐SEM data G) Enlarged segmentation from black square from F, showing a splitted nucleus during nuclear relocalisation in the final stages of ookinete development. H) Mitochondrial tunneling of nuclear appendices from SBF‐SEM data.

Next, we assessed if the nucleus, as the entire cell, also undergoes developmental changes. These potential subcellular developmental changes could reveal unique adaptations specific to the different needs of the developing ookinete and, in addition, could reveal additional subcellular hallmarks of development. During ookinete development, the nucleus undergoes a single meiotic DNA replication without performing karyokinesis, resulting in a tetraploid nucleus.^[^ ^28^ ^]^ Targeted vEM acquisition allowed us to investigate nuclear morphology and positioning in the cell. This revealed an overall spheroidal shape that changes in relative cellular positioning as the nucleus repositions from the zygote into the emerging ookinete (**Figures** **4**A, s3 A, Supporting Information). Intriguingly, nuclear volume is reduced over time from 10 µm^3^ to 5 µm^3^ (Figure 4B). This follows in line with overall cellular volume reduction and might be also due to changes in transcriptional activity. Curiously, the nuclear to cytoplasmic ratio appeared to decrease with development (Figure S3E), while in blood stages this ratio increases.^[^ ^29^ ^]^

We found that nucleoli appeared during stage II and were present until the end of development, with a mean volume of roughly 0.02 µm^3^ (Figures S3B and S4, Supporting Information). Additionally, the intranuclear space contained electron‐dense stripes, the synaptonemal complexes, indicative of meiosis, during the early stages (Figure 4A, light green & Figure S4, Supporting Information), which reduced in volume over the course of ookinete development (Figure S3C, Supporting Information).^[^ ^14^ ^]^ In stages I to V, the nuclei were positioned in the bulb and in stages I – III showed several types of protrusions from the nuclear envelope, which we termed tails and appendices. Appendices are convoluted membrane protrusions contrasting with the straight tails that appeared from 500 nm to several microns in length (Figure 4A, Figure S4, Supporting Information). ssET of a nuclear tail revealed a single microtubule at the interior (Figure 4C) that likely originates from a centriolar plaque (CP), the spindle pole body like structure in *Plasmodium* (Figure 4D).^[^ ^24^, ^30^, ^31^ ^]^ While SBF‐SEM readily revealed nucleoli, FIB‐SEM also allowed the detection of CPs and ssET, in addition, revealed microtubules (Figure 4E). We observed four CPs per nucleus arising at Stage VII. Intranuclear microtubules (INMTs) emerged from the CPs and ranged from 100 nm to 800 nm in length, with a mean length of ≈400 nm in the fully mature ookinetes (stage VII) (Figure 4D). Compared to INMTs in asexual blood stages of *P. falciparum*, the INMTs in ookinetes were on average approximately twice as long.^[^ ^31^ ^]^

In stages V to VII the nuclei adopted a more oval shape and were repositioned to the center of the cell in the mature ookinete (Figure 4F,G). In two of the late stages (one from SBF‐SEM, one from FIB‐SEM), we also observed the separation of the nucleus into two nuclei during its relocalisation (Figure 4G, Figure S3G,H, Supporting Information). The ssET dataset revealed 34 nuclear pores that were clustered at one side of the nucleus (Figure S3F,G, Supporting Information). 26 nuclear pores appeared on the surface of the nucleus closest to the IMC and in the vicinity of the centriolar plaques. A rosette arrangement of nuclear pore complexes (NPCs) surrounding the centriolar plaque as seen using U‐ExM was not observed.^[^ ^32^ ^]^ The pores were at a distance of 150 to 730 nm to the IMC. No nuclear pores were found at the surface facing the apical end, similar to what was observed in blood stages.^[^ ^5^, ^33^ ^]^ In contrast, the nuclear appendices showed no particular orientation, but deep invaginations in all ookinetes at stages III and IV (Figure 4A,H and Figure S4, Supporting Information). These invaginations were associated with tubular mitochondria that tunneled through the appendices (Figure 4H). This mitochondrial tunneling was seen in all nuclei that possessed nuclear appendices, suggesting some level of functional relevance.

### 2.5 Mitochondria are Pleomorphic with Variable Distribution During Ookinete Development

**Figure 5:**
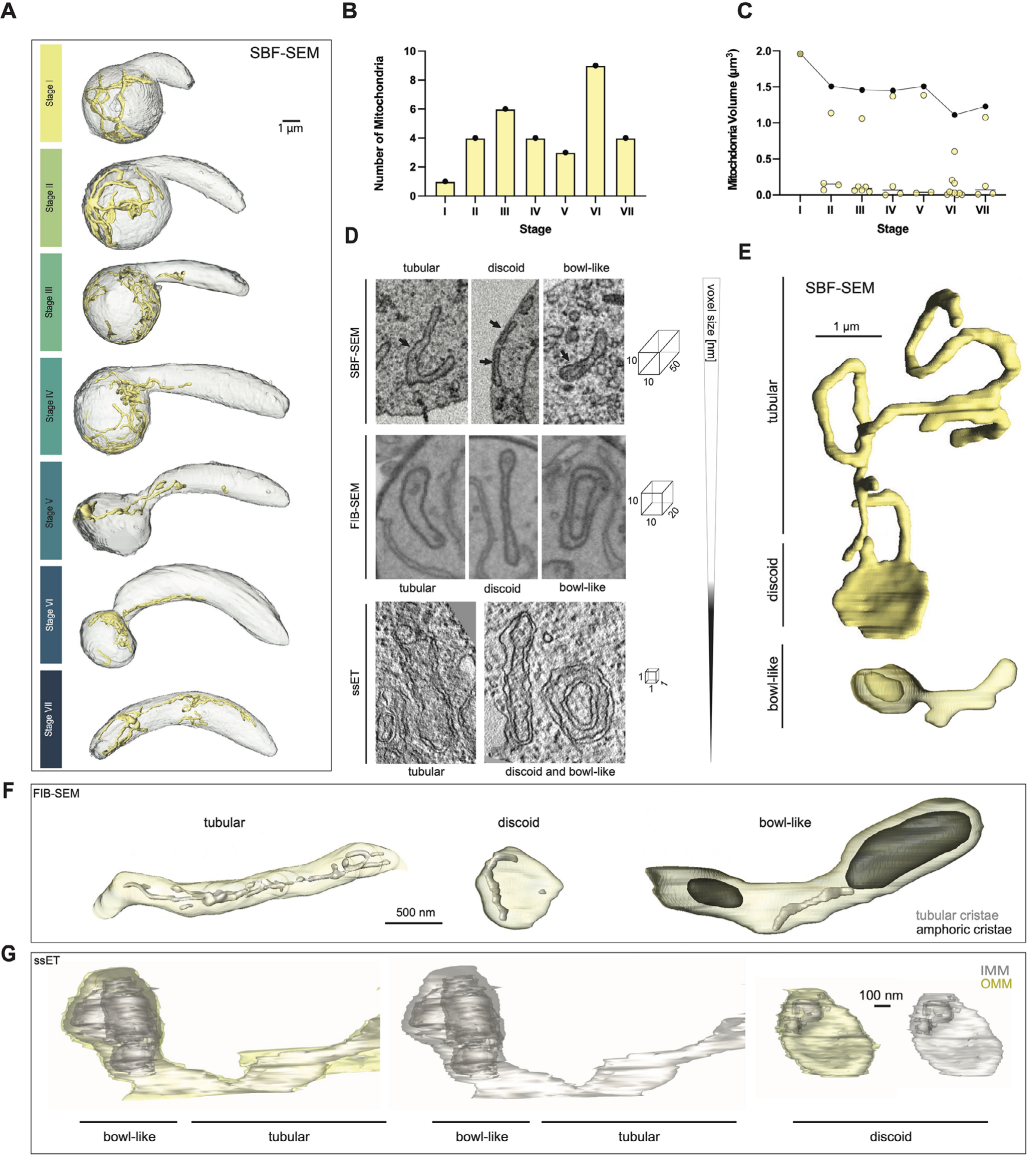
Mitochondria distribution, volumes and ultrastructural details during ookinete development. A) Full mitochondria segmentation of one cell of each stage. B) Number of mitochondria present in the ookinetes shown in A and sorted by stage. C) Volume percentage of all (black) and individual (yellow) mitochondria in the ookinetes shown in A. D) Single slices of mitochondria using three vEM techniques with varying voxel sizes as indicated, revealing different ultrastructural details. E–G) Volume reconstructions of mitochondria with different morphological types from SBF‐SEM (E), FIB‐SEM (F) and ssET (G). Note the presence of different types of cristae (tubular and amphoric); IMM: inner mitochondrial membrane, OMM: outer mitochondrial membrane.

Upon passage to a mosquito host, the parasite's metabolism transitions from glycolysis to oxidative phosphorylation rendering mitochondria a key organelle of interest. Mitochondria form a network in different stages of *Plasmodium*.^[^ ^34^, ^35^ ^]^ In ookinetes, 3D EM imaging allowed us to distinguish between one and nine mitochondria that form a network throughout the cell (**Figure** **5**A,B). Of these multiple mitochondria, one large mitochondrion contributed to the majority of the mitochondrial volume (50–100%), while the remaining mitochondria were much smaller. Altogether, the total mitochondrial volume ranged between 1 and 2 µm^3^ (5% of the total cell volume) across all stages of ookinete development (Figure 5C). Whether the change in number reflects a true dynamic change or the small number of cells observed needs to be investigated. As the retort elongated to form the stereotypical mature ookinete morphology, the mitochondrial network transitioned from a cortical localization abutting the PM in the bulb to a more diffuse distribution throughout the whole cell. During stages I‐III, the mitochondria resided in the bulb of the retort. As the cell developed further (stages III to VI), the mitochondrial network migrated to the distal end of the bulb, close to the opening of the protrusion. Then, at the end of development (stage VII), the mitochondrial network could be found throughout the ookinete. The mitochondria never occupied the apical tip of the growing retort's protrusion (stages I‐VI) and were also absent from the apical part of the mature ookinete (Stage VII), only occupying the posterior 80% of the cell (Figure 5A). Our vEM analyses revealed three main mitochondrial morphologies: tubular, discoid, and bowl‐like (Figure 5D–G), which were all found at every stage of development. We found that the tubular morphology appeared to be the most abundant, followed by bowl‐like, and then discoid and various other mitochondrial morphologies (Figure S5, Supporting Information).

FIB‐SEM and ssET data enabled characterization of the inner structures of the mitochondria with enhanced resolution, and two different membrane invaginations were identified. We observed tubular cristae with a single membrane and amphora‐like cristae with an apparent double membrane (Figure 5D,F). Additionally, electron tomography revealed that the double membranes of the mitochondria have electro‐lucent intermembrane spaces and a wide range of cristae morphology has been observed. (Figure S 5). These changes might accompany the transition from glycolysis to oxidative phosphorylation.

### 2.6 Elongated Micronemes Interact with the Apicoplast but Rarely with Microtubules

**Figure 6:**
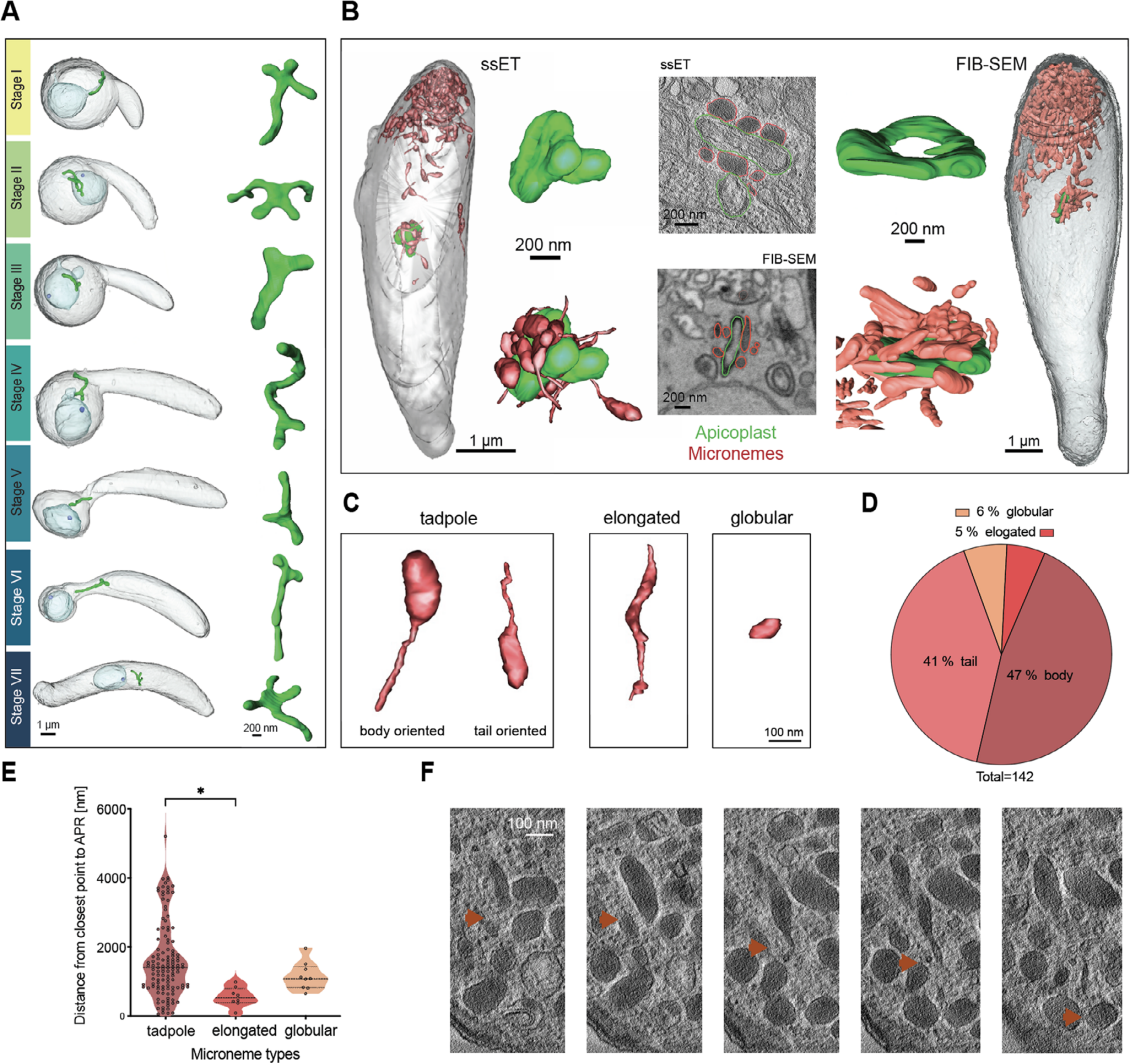
Micronemes are elongated and interact with the apicoplast. A) Representative segmentation of an apicoplast within the full cell and the nuclei to visualize organelle topology and close up views of segmentations of each apicoplast. B) Full cell and single slice representations with apicoplast and microneme segmentation from FIB‐SEM and ssET. Note close association between apicoplast and micronemes. C) Segmentations of different morphological types of micronemes observed in ssET. D) Relative distribution of different microneme types in a single ookinete. E) Distance of each microneme to the apical polar ring measured from ssET data. *: *p* < 0,05 (Student's *t*‐test). Horizontal bars represent the mean, 25 and 75 percentiles. F) Single slices through a “tadpole” microneme (red arrow).

Apicoplast and micronemes are parasite‐specific organelles important for core metabolic functions, such as lipid biosynthesis, and for secretion of proteins involved in cell migration, respectively.^[^ ^36^, ^37^ ^]^ With SBF‐SEM, we were able to differentiate the apicoplast from the mitochondria by its denser and thicker membrane coat, which consists of four membranes, and its more electron‐lucent lumen (**Figures** **6**A, S6A, Supporting Information, green arrows).^[^ ^36^, ^38^ ^]^ The ssET data revealed the four membranes, although in some datasets only three membranes could be resolved (Figure S6E,F, Supporting Information). Over the course of ookinete development, the apicoplast retained a tube‐shaped morphology with some being branched (Figure 6A). We found these branches either at the side, where the apicoplast was attached to the nucleus or at the side facing the opening of the protrusion (Figure 6A, Figure S6, Supporting Information). It is unclear whether the two organelles are physically tethered, which could be investigated with cryo‐fixed samples. The apicoplast in the mature ookinete consisted of a discoid center, which could be branched or unbranched (Figure 6A,B, Stage VII; Figure S7, Supporting Information). While the apicoplast volume decreased over the course of development, from 0.14 to 0.08 µm^3^, the surface area stayed ≈2.5 µm^2^ and there seemed to be no correlation between the number of branches and the stage of the ookinete (Figure S6B–D, Supporting Information). The apicoplast data was not included in the cluster analysis for parasite staging as the observed small differences did not provide stage‐specific information.

In contrast, the alterations to the subcellular localization and morphology of the micronemes featured in the cluster analysis. When investigating the micronemes across all datasets, two major populations of micronemes were found, one located at the ookinete's apical end and the other surrounding the apicoplast (Figure 6B,E). In stages VI & VII, the more discoid‐like apicoplast was surrounded by micronemes (Figure 6B). We identified three distinct types of micronemes: tadpole‐shaped micronemes with strikingly long tails, elongated micronemes with a uniform diameter, and globular micronemes (Figure 6C). The vast majority, 88%, of the 142 micronemes analyzed from the ssET dataset appeared tadpole‐shaped with a length of 750 nm and a maximal width of 150 nm, while 6% were globular (diameter of 100 nm) and 6% elongated (length of up to 750 nm and width of 50 nm) (Figure 6D). Fourteen micronemes were in close association with the apicoplast. All micronemes together accounted for 1.2% of the ookinete volume. Measuring the distance from the apical end to the micronemes revealed that the elongated micronemes localized closer to the apical end than the tadpole shaped ones (Figure 6E). 58 of the tadpole‐shaped micronemes (41%) showed their elongated tail pointing toward the apical tubulin ring while 67 (47%) pointed in the opposite direction. Previous work showed close association of micronemes to microtubules.^[^ ^39^, ^40^ ^]^ Surprisingly, we found only 16 (out of 142) micronemes located in proximity (distance < 50 nm) to microtubules, while most of them were not associated with microtubules with an average distance between micronemes and microtubules of 260 nm (Figure S8, Supporting Information). We also found no correlation between the distance of micronemes to microtubule and the distance of micronemes to the apical tip (Figure S8, Supporting Information). The different microneme morphology may reflect different stages of maturation or different populations of micronemes with distinct functions or might even be different types of vesicles.

### 2.7 Reconstructing the Sub‐Pellicular Microtubule Cytoskeleton

**Figure 7:**
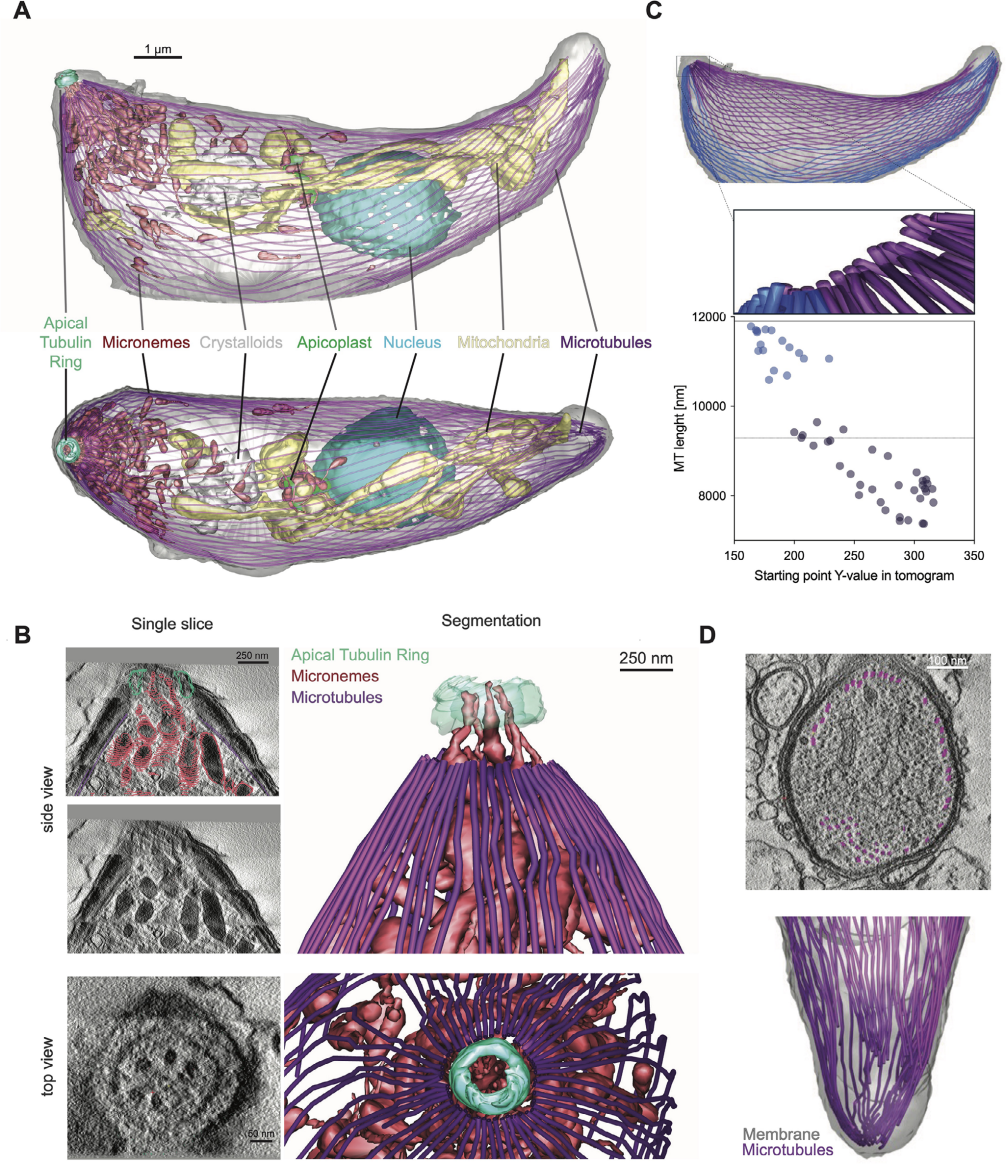
Full‐cell segmentation of subpellicular microtubules from ssET data. A) Top and side view of a whole cell segmentation with indicated organelles B) Single slice and whole volume views of microneme arrangement at the apical end of the ookinete, showing threading of elongated microneme protrusions through the apical tubulin ring. C) Microtubules at the concave side (violet) are shorter than on the convex side (blue) of the ookinete. D) Microtubule dissociation from the IMC at the distal end of the ookinete.

The ssET dataset allowed the nearly complete tracing of all microtubules in a stage VII ookinete from the apical to the distal end (**Figure** **7**A,C). This revealed at the apical tip, the apical tubulin ring or conoid (Bertiaux et al., 202 1^41^) with an outer diameter of 300–350 nm, an apparent thickness of 60–70 nm and a height of 140 nm (Figure 7B).^[^ ^41^ ^]^ The conoid was located at a distance of 120 nm to the presumed apical polar ring where microtubules originated. Five microneme tails could be distinguished threading through the aperture of the conoid (Figure 7B). Four of these were tadpole shaped micronemes and one showed an elongated shape. In this way multiple micronemes reached the apical end of the ookinete and could, hence, be primed for fusion with the plasma membrane and secretion of their content. At the apical end, 53 microtubules started, presumably at the apical polar ring.^[^ ^26^, ^41^ ^]^ The polar ring itself, however, was not distinguishable from the apical collar, similar to tomograms from FIB milled lamellae.^[^ ^42^ ^]^ Of all 53 microtubules, 40 could be completely traced until the distal end with a length of 7.3 to 11.7 µm (average 9.2 µm). Two other microtubules stopped after 650 nm and the remaining eleven were interrupted by missing volume, yet, they could be interpolated as all of them continued after the gap (Figure S9, Supporting Information). The microtubules originating at the concave side of the cell were significantly (*p* < 0.0001) shorter compared to those originating and extending along the longer convex side of the parasite (Figure 7C). Interestingly, all microtubules reached far into the narrowing basal end, which caused some to lose their association with the IMC (Figure 7D). From these measurements and the volume of the entire ookinete, the concentration of polymerized tubulin was calculated to be 44 mM, which is ten times the concentration of what is needed to facilitate polymerization of tubulin in vitro.^[^ ^43^ ^]^

### 2.8 A Subcellular Atlas of Ookinete Development Reveals Changes to Cellular Morphology, Size, Organelle Structure and Localization

**Figure 8:**
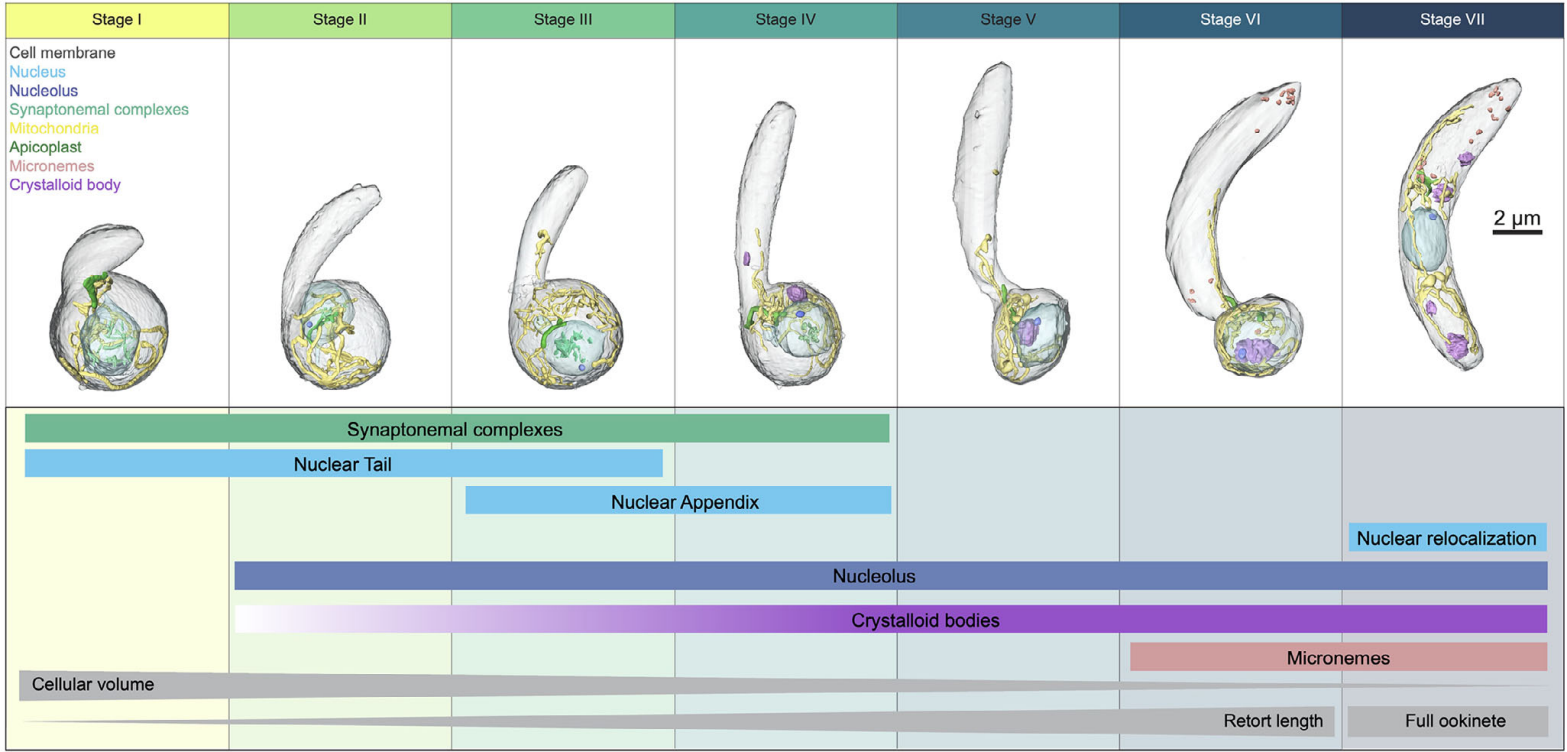
Cellular hallmarks of ookinete development. Recapitulation of whole cell ookinete development along all identified stages with cellular hallmarks noted inside the box.

A full segmentation of one representative cell for each stage is shown in **Figure** **8**, capturing the cellular features and characteristics over the course of ookinete development. We classified, in an unbiased way, seven distinct developmental stages based on cellular features, with the newly identified nuclear morphologies being especially signatory of the specific stages. We show that the presence of synaptonemal complexes spans from Stages I to IV, which indicates that meiosis occurs in these stages. Nuclear tails are present from Stages I to Stage III, coinciding with nuclear appendices from Stages II to IV, and then in the final stage VII, nuclear relocalisation occurs, with the nucleus moving from the bulbous end of the retort to the middle of the mature ookinete. Beyond nuclear morphology, our data shows that the onset of crystalloid body formation occurs already at Stage V, while their abundance increases over the course of development. Additionally, we see that micronemes form in Stage VI at the bulb of the retort and are relocated to the apical pole at the end of development in Stage VII. Furthermore, cellular volume decreases over the course of development as the length of the retort protrusion increases (Figure 8).

## 3 Discussion

Developmental transitions of cells are essential for both protozoan and metazoan organisms and currently most frequently studied on a transcriptomic or proteomic level.^[^ ^44^, ^45^, ^46^ ^]^ However, these lack visual subcellular resolution. Here we explored the use of three vEM methods to study developmental transition using the ookinete stage of *Plasmodium* as model cell. Ookinetes are elongated motile cells that develop from round zygotes over several hours, transitioning through a series of morphologically distinct stages. Of the many possible developmental transitions of *Plasmodium* to study we chose the development of ookinetes for a number of reasons. Like any other stage transition, ookinete development is essential for parasite life cycle progression. Ookinetes can be easily cultured and follow a clear developmental path that to date was not well defined. Using SBF‐SEM, we acquired 28 cells at different stages of development and determined key morphometric parameters of organelles to generate an unbiased cluster analysis that allowed us to stage the developmental transitions (Figure 8). To support and extend from this analysis we also imaged 33 ookinetes by FIB‐SEM and reconstructed an entire mature ookinete at high resolution by ssET. These datasets provide an open access subcellular atlas that will inform functional studies. The multitude of cellular hallmarks allowed us to subdivide the continuous ookinete development into seven distinct stages.

As vEM has the capacity to delineate cellular developmental transitions, it's application will enable new ways to investigate morphogenesis. vEM techniques have also been used to investigate various cellular processes in both model cells and cells of medical interest.^[^ ^52^, ^53^ ^]^ Parasites are of medical interest because they infect hundreds of millions of people every year and cause significant harm, and of fundamental biological interest due to their highly divergent nature compared to classic model eukaryotes. vEM has recently also been applied for the study of protozoan parasites including those causing sleeping sickness, Chagas disease, leishmaniasis, cryptosporidiosis, toxoplasmosis and malaria.^[^ ^47^, ^48^, ^49^, ^54^, ^55^, ^56^, ^57^, ^58^, ^59^, ^60^ ^]^ These have all revealed interesting new insights into organelle structure, cytoskeletal arrangements and stage transitions. Other developmental transitions in *Plasmodium* have already been studied by whole cell vEM showing that the approach can be widely applied but no cluster analysis of the obtained data could be performed, due to the small set of imaged cells.^[^ ^5^, ^35^, ^47^, ^48^, ^49^ ^]^ Here we expand on these approaches by introducing unbiased cluster analysis for developmental staging, combining different vEM methods bridging high throughput and high resolution as well as by providing all data in the original resolution as open access files that can easily be browsed using image data explorer or downloaded for further and comparative analysis (SBF‐SEM data: EMPIAR ID‐12951, ssET data: EMPIAR ID‐12955, FIB‐SEM data: EMPIAR ID‐12956).^[^ ^61^ ^]^ Future work could also incorporate ultrastructure expansion microscopy (U‐ExM) in this unbiased analysis approach, a complementary higher throughput but lower resolution technique used to image developmental progression of blood and mosquito stage parasite through localization of specific protein and lipid structures using fluorescent antibodies and stain.^[^ ^41^, ^50^, ^51^ ^]^

Despite the reported advancement, our study also has limitations. All our preparations were done using classic fixation techniques, which can lead to artefacts such as membrane distortions and fusions. These limitations could be overcome by using cryo‐fixation methods such as high pressure freezing followed by freeze substitution. Despite investigating over 60 different cells, allowing us to delineate a pseudo‐time development, a much higher number of imaged cells could reveal more “temporal” detail and inform on some of the highly dynamic processes. We used different clustering methods and obtained between 5 and 7 different stages depending on the method and the threshold we used and as such these stages should be considered as fluidly moving from one to another. Most clustering methods will leave some level of ambiguity where to split closely clustering groups into two.

Recording 28 cells by SBF‐SEM took 2 full days and recording the dataset including 33 cells by FIB‐SEM took over a week. However, the current bottlenecks are the segmentation of the vEM datasets to extract organelle shapes and the determination of the presence or absence of a subcellular feature. These bottlenecks will likely be overcome in the near future by the development of automated AI‐based segmentation tools.^[^ ^62^, ^63^ ^]^ In anticipation of these new segmentation tools, we freely provide our data for future analysis of features that we did not delineate such as, for example, the endoplasmic reticulum or that we delineated only at a representative scale such as mitochondria. Such faster analysis methods will then provide the basis for higher throughput studies of parasites that have been genetically modified, which will enable investigation of genetic defects to ultrastructural alterations. To do so would not necessarily need the imaging of the entire cell but could be limited to subcellular sections, thus again speeding up analysis. We thus predict that soon the comparative analysis of hundreds of cells will be possible at affordable cost and complement ongoing efforts in phenotypic high throughput screens in *Plasmodium* and other organisms.

Subcellular vEM was used in several studies to resolve microtubules, which are essential for many stage progressions.^[^ ^30^, ^64^, ^65^ ^]^ Microtubules were also shown to be essential for ookinete formation.^[^ ^66^ ^]^ We opted to examine microtubules with ssET, which is comparatively labor intense during preparation, recording and the reconstruction of, in our case, 57 tomograms from 23 serial sections. Compared to U‐ExM data our dataset adds a number of interesting observations.^[^ ^20^, ^41^ ^]^ For example, we provide the first complete measurement of the conoid, which has been discovered by U‐ExM and partially visualized at high resolution from FIB‐milled lamellae using Cryo‐ET.^[^ ^41^, ^42^ ^]^ We found two “subsets” of subpellicular microtubules with different lengths aligning with the concave and convex side of the parasite and found that microtubules dissociate from the IMC at the distal end of the ookinete. This indicates that the parasite might not have a mechanism to stop microtubule growth and makes them simply as long as it can until they run out of space. Most importantly, our dataset reveals that micronemes are largely not associated with microtubules, but showed a tight association with the apicoplast. Indeed, similar numbers are found associated with both microtubules (16/142) and the apicoplast (14/142). But naturally, our data gives no insights into the dynamics of potential microtubule transport or the duration of apicoplast association. Also, maturation of micronemes might occur or micronemes might be present in different subpopulations as suggested by their different shape. The tight association with the apicoplast could hint toward a direct transfer of lipids, some of which are generated in the apicoplast, to the micronemes. However, this hypothesis would need to be tested by lipidomics. Intriguingly, a recent report showed that a micronemal protein was important for apicoplast biogenesis in the liver stage suggestive of the existence of some organellar crosstalk.^[^ ^67^ ^]^ Micronemes in the ssET dataset appeared as unexpectedly elongated vesicles with the vast majority exhibiting a long tail‐like protrusion that could not be detected with the other methods. These protrusions might be functionally relevant for apical membrane fusion. Such protrusions could either form by pulling the membrane from outside or by pushing from within the vesicle. In all other examined parasites including *Eimeria*, *Cryptosporidium*, *Toxoplasma* and other *Plasmodium* stages only ovoid micronemes were found.^[^ ^57^, ^68^, ^69^, ^70^ ^]^ Intriguingly, ookinetes are the only motile parasite stage that do not show rhoptries, elongated secretory organelles that are important for host cell invasion.^[^ ^71^ ^]^ The thin protrusions of rhoptries, called rhoptry necks, show internal spiral filaments, which are hypothesized to shape the rhoptries.^[^ ^72^ ^]^ Similar filaments might well be present in the long protrusions of micronemes in ookinetes but would necessitate cryo‐electron tomography for detection.

The nuclei of ookinetes also showed protrusions, which could be classified into two different categories, tails and appendices. The tails are most likely produced by polymerizing microtubules pushing the nuclear envelope from inside the nucleoplasm (see also Hentzschel et al., 202 3^24^), as has also been seen in nuclei during sporozoite formation and in schizonts.^[^ ^24^, ^30^, ^31^, ^64^, ^73^ ^]^ The formation of the appendices is enigmatic, as well as their intimate association with mitochondria that appear to tunnel through the nucleus. A close interaction of nuclei and mitochondria has been observed in different apicomplexans and *Plasmodium* stages.^[^ ^70^, ^74^ ^]^ However, the tunneling found in ookinetes appears unique. We speculate that it might ensure rapid transport of energy either into the nucleus for meiosis or into the nuclear envelope to enlarge the ER and aid generation of secretory vesicles.

Mitochondria showed interesting polymorphic forms with three distinct shapes, which were partly described using TEM of ookinetes and FIB‐SEM of blood stage parasites.^[^ ^13^, ^74^ ^]^ Potential functional differences between these three mitochondrial morphologies remain speculative. When the electrochemical gradient of human mitochondria is depolarized in cell culture, mitochondria adopt similar rounded, discoid shapes with several infoldings of the inner membrane.^[^ ^75^ ^]^ This suggests that the bowl‐like and discoid invaginations of ookinete mitochondria might be less efficient in energy production. Indeed, energy production via the respiratory chain is most important for later oocyst stages.^[^ ^34^, ^76^ ^]^

While SBF‐SEM mapped mitochondrial localization, FIB‐SEM revealed the intraorganellar ultrastructure, displaying two distinct types of inner membrane invaginations: tubular and amphoric cristae (Figure 5D). These structures were shown to be interconnected within the mitochondria. FIB‐SEM data revealed that the tubes have a single membrane, whereas bowls have a double membrane. However, FIB‐SEM alone could not clarify how the double membrane is formed. Using ssET, we segmented the mitochondria, showing that the bowl‐like structures are 3D infoldings of the inner mitochondrial membrane, rather than a separate third membrane. Similar findings were supported by Cryo‐ET data from Ferreira et al., (2023).^[^ ^42^ ^]^ These bowl‐like structures often have multiple infoldings (Figure 3F,G).

The apical pellicular membrane folds have been observed before in various studies using TEM and SEM, but have been left mostly uncommented.^[^ ^20^, ^77^ ^]^ Pellicular folds are common in gregarines, where they can either point longitudinally, or, like in ookinetes, perpendicular to the parasite long axis.^[^ ^78^ ^]^ Here, we observed a mean of 3.6 folds per ookinete with 90% of stage VII ookinetes showing at least one of these folds. In these folds, the plasma membrane and the IMC bulge and extends up to 200 nm away from the underlying SPMTs. As these folds are only observed in late stage ookinetes they might have a function in or be a result of active gliding motion. In apicomplexans, the IMC is linked to the SPMTs by a number of proteins.^[^ ^42^, ^79^ ^]^ This linkage is thus either ruptured below the folds or the linking proteins might not localize at sufficient concentration to the region where the folds can be observed.

Finally, we observed a curious contraction of the ookinete volume throughout its development, while the surface area is static at ≈120 µm^2^. In other apicomplexa parasites the cell volume is doubling throughout development.^[^ ^56^ ^]^ We speculate that the decrease in volume involves osmotic regulation and arises from the necessity to pack all the cellular components for successful transmission in a confined space.

In conclusion, our study provides a proof‐of‐concept methodology that enables the unbiased analysis of cellular developmental processes by vEM. Our study also provides the basis for allowing a combination of vEM data with transcriptional, reverse genetic or proteomic profiling data and provides an ultrastructural atlas onto which future work can map morphological changes of mutant parasites. Importantly, our study, has also general implications for developmental cell biology far beyond *Plasmodium* research. Our unbiased cluster analysis and data‐driven approach, effectively mitigates the ambiguities associated with traditional staging methods, allowing for a more accurate representation of cellular developmental progression in any organism.

## 4 Experimental Section

### Ethics Statements

Animal experiments were performed according to FELASA and GV‐SOLAS guidelines and approved by the responsible German authorities (Regierungspräsidium Karlsruhe). *Plasmodium* parasites were maintained in 5‐ to 8‐week‐old female Swiss mice obtained from JANVIER. Animal research at Umeå University was conducted under Ethics Permit A13‐2019 and approved by the Swedish Board of Agriculture. Female BALB/c mice used at Umeå University were purchased from Charles River Europe.

### Preparation of Ookinetes for SBF‐SEM

Ookinetes were produced using the *P. berghei* line Pb473, a *P. berghei* ANKA line that constitutively expresses RFP, a kind gift from Drs Andy Waters and Katie Hughes (University of Glasgow). Two female Swiss mice were injected intraperitoneally with 200 µl phenylhydrazine (6 mg mL^−1^ in PBS) to stimulate reticulocytosis. 2 days later, the mice were infected intraperitoneally with 20*10^6^ iRBC Pb473. Mice were bled 3 days post infection and 500 µl blood was transferred to 10 ml ookinete medium (RPMI supplemented with 20% (v/v) FCS, 50 µg mL^−1^ hypoxanthine, and 100 µM xanthurenic acid, adjusted to pH 7.8 – 8.0) at 19 °C. After 22 h of culture, ookinete cultures were underlayed with 5 ml 55% Nycodenz/PBS and centrifuged for 25 min at 1000 rpm without brake. The interphase containing purified ookinetes was collected, washed once in PBS and immediately processed for EM.

### Sample Preparation and Correlative Targeting for SBF‐SEM

To facilitate a detailed ultrastructural analysis of ookinete development the throughput of SBF‐SEM imaging needed to be increased, and for this a light microscopy targeting method was employed: First purified gametocytes were transferred to ookinete medium and after certain time points post fertilization the cells were adhered to gridded (MatTek) dishes, that contain coordinates for correlative experiments using poly‐L‐lysin. Cells were fixed in 2.5% glutaraldehyde (EMS) and 2% paraformaldehyde (EMS) in 0.1 M sodium cacodylate buffer for 30 min and stained with DAPI for subsequent screening and targeting of cells by light microscopy. Next, the cells were imaged using a wide‐field light microscope (Zeiss) and ROIs on the dish were selected for containing several cells of interest based on their morphology, DAPI staining and cytoplasmic RFP (Figure 1C). After light microscopy targeting, the cells were prepared for electron microscopy imaging, which was assisted using a PELCO BioWave Pro+ (Ted Pella). All incubation steps were performed inside the BioWave for 14 min total with each 2 min on/off cycles at 150 W, with vacuum and two rinsing steps for 40 s at 250 W and no vacuum. Post‐fixation was performed in 1% OsO4 reduced with potassium ferrocyanide (0.8%) in 0.1 M cacodylate buffer, followed by 1% aqueous tiocarbohydrazide and a subsequent 1% aqueous OsO4 incubation. Dehydration was performed with a graded ethanol dehydration series of 10% increments (10% to 100% ethanol) inside the BioWave for 40 s each at 250 W with no vacuum. Samples were embedded in EPON with a graded series (10%, 25%, 50%, 75% and 3×100% EPON) using a BioWave with 3 min for each step of the graded series (250 W and no vacuum). The resin was cured at 60 °C for 48 h and the resulting blocks were re‐imaged by transmitted light microscopy (Zeiss) confirming the presence of the ROIs of interest at the corresponding coordinates transferred to the bloc surface (Figure 1D). The trimmed blocks were cut down to a size of roughly 500×500 µm either manually using a razor blade, or using an ultramicrotome (Leica, UC7) with a 90° trimming knife (Diatome) and glued to a SBF‐SEM aluminum pin (Micro to Nano) and subsequently transferred to the SBF‐SEM system, consisting of a Zeiss Gemini 2 SEM equipped with a Gatan 3view microtome. The microscope was operated using Zeiss Smart SEM software and SBEM Image (Titze et al., 2018), to acquire a block overview and high‐resolution images of the ookinetes (Figure 1E). The overview was used to identify the regions of interest and to place the high‐resolution imaging tiles, speeding up acquisition once more. The SEM was operated at 1.5 kV and 500 pA with a pixel dwell time of 1.6 µs. Focal charge compensation was used to mitigate charging artifacts. The cutting thickness was either 40 or 50 nm and the frame size of the high‐resolution frames was 2044×1536 pixels.

### Ookinete Enrichment and Sample Preparation for FIB‐SEM

Phenylhydrazine‐treated mice were infected with the Pb473 strain of *P. berghei* strains. Infected blood samples were harvested on day 3 post‐infection and further cultivated in the ookinete culture medium at 19 °C to obtain ookinetes at different developmental stages (Ramakrishnan et al., 2012). The culture was harvested and washed in PBS at 500 × g, 4 °C. Ookinetes were then further enriched in 17.3% Nycodenz at 500 × g, 4 °C without a full break to slow the centrifugation speed down. The enriched band above Nycodenz was collected and washed in PBS at 500 × g, 4 °C. The sample was further enriched using the LD column (Miltenyi) according to the manufacturer's protocol. The plunger provided with the column was used to elute the parasite. Parasite samples were fixed in 2.5% glutaraldehyde (Sigma‐Aldrich) in 0.1 M PHEM buffer and processed following the preparation of biological tissues for serial block face scanning electron microscopy protocol version 2 without lead aspartate staining (Deerinck et al., 2022).

### Sample Preparation for Electron Tomography

Ookinetes were fixed in 2% glutaraldehyde and 2% paraformaldehyde in 0.1 M cacodylate buffer and post‐fixed with 1% OsO4 for 1 h and were incubated overnight at 4 °C in uranyl acetate. Samples were dehydrated in a graded series of aqueous acetone (30%, 50%, 70%, 90%), followed by two additional 10 min dehydration steps with 100% dried aceton solution. Samples were embedded ins Spurr's resin (23.6% (ERL), 14.2% ERL‐4206 plasticizer, 61.3% nonenyl succinic anhydride and 0.9% dimethylethanolamine) in a graded series (25%, 50%, 75%) for 45 min each. After overnight incubation at 4 °C in 100% spurr's resin, the sample polymerized overnight at 60 °C. 200 nm thick sections were acquired using a Leica UC7 ultramicrotome and transferred on copper grids. Tomograms were acquired using a Tecnai F20 TEM (FEI) at 200 keV operated with SerialEM (Mastronade, 2003). Tilt series were acquired at 9600× magnification from −60° and +60° with a 2° increment. Image alignment, reconstruction and segmentation was performed in IMOD 4.11 (Kremer et al., 1996).

### Statistical Analysis

Segmentation of SBF‐SEM datasets and quantification of features was done in Amira and Fiji. Spectral seriation, UMAP, PCA analysis, hierarchical clustering and visualization (Figure 2A–D) were performed in R 4.3.0. The features of cellular volume and lengths of the cell protrusions were assessed as a continuous variable, the number of cristalloids was counted, while all the other features were logical variables, that is, true or false. For the UMAP, the size of local neighborhood parameter was set to 5. For spectral seriation and hierarchical clustering, the gower's distance was used to ensure reliable results for all variable classes. For hierarchical clustering and PCA, the features were scaled to a mean that equals zero, and a standard deviation of one (logical variables were transformed to 0 and 1). For visualization in Figure 2E, all features were scaled to a total range of 1, with their minimum value being zero while the maximum was one. Data is presented with all absolute values as dot blots. Sample size is stated in the figure legends with n = 3 volumes of different cells for each proposed stage. Statistical tests are mentioned in the figure legends. For statistical analysis GraphPad Prism (v.10.2.3) was used.

## Conflict of Interest

The authors declare no conflict of interest.

## Supporting information



Supporting Information

## Data Availability

The data that support the findings of this study are openly available in Image Data Explorer at https://shiny‐portal.embl.de/shinyapps/app/01_image‐data‐explorer?settings═https://s3.embl.de/screens/IDE_config‐plasmodium_ookinetes_data.toml, reference number 47486685.
