# Supplementary material for: Cellular Hallmarks From Volume Electron Microscopy Reveal Developmental Progression of Plasmodium Ookinetes
Source: Adv Sci (Weinh). 2025 Sep 30;13(4):e08250. doi: 10.1002/advs.202508250 (PMC12822446; doi:10.1002/advs.202508250)
Supplement: Supplementary file 1 — Supporting Information [file ADVS-13-e08250-s001.docx]

Supplementary figures


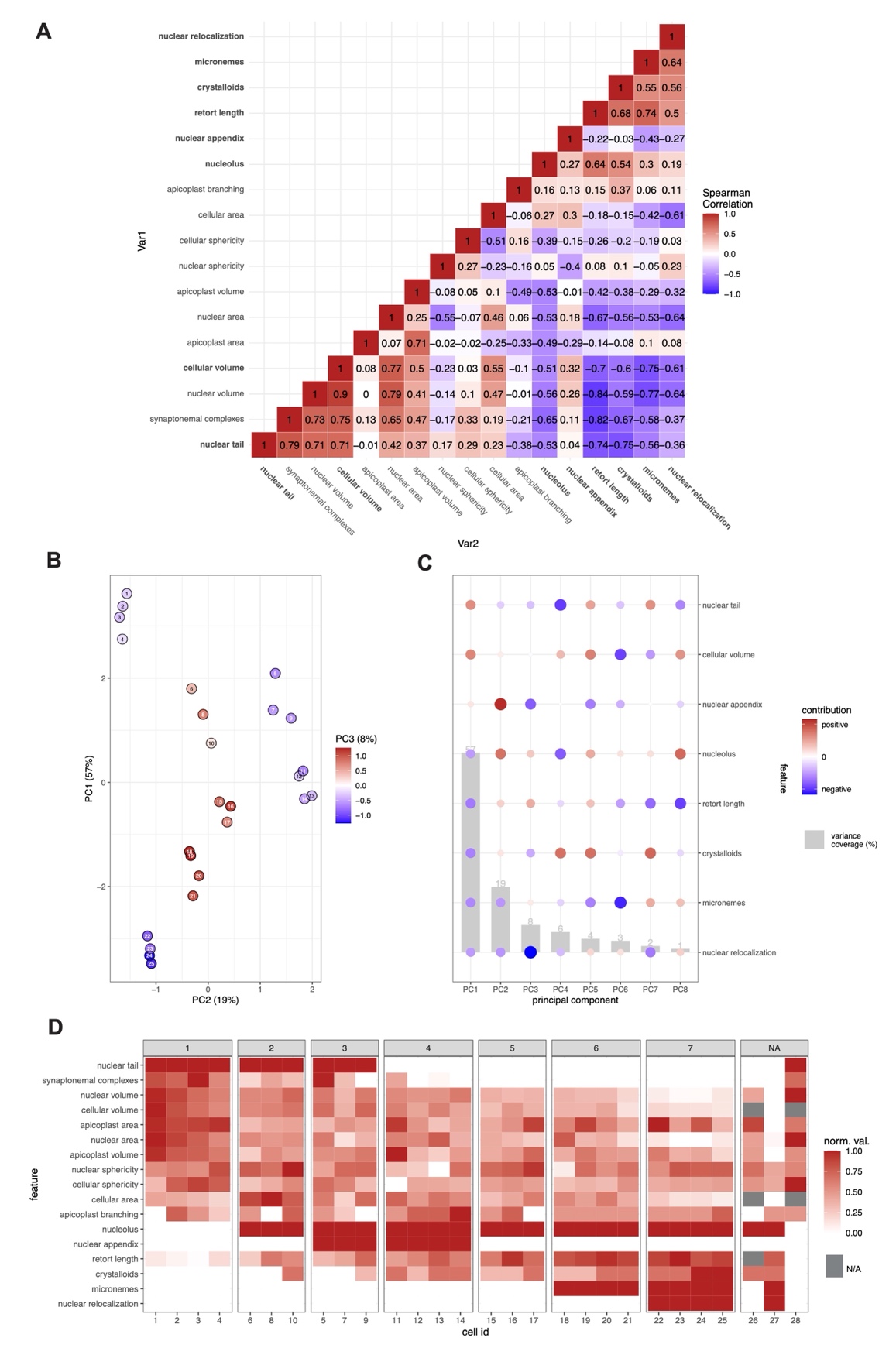


**Supplemental Figure 1: Clustering analysis and heat map of morphometric features.**

A: Cross-correlation analysis of all factors analysed, chosen parameters for cluster analyses are indicated in bold.

B, C: PCA Analysis of SBF-SEM data (B) and each pricipal component contribution (C).

D: Heat map of all features analysed. Normalised values are either presence or absence of cellular


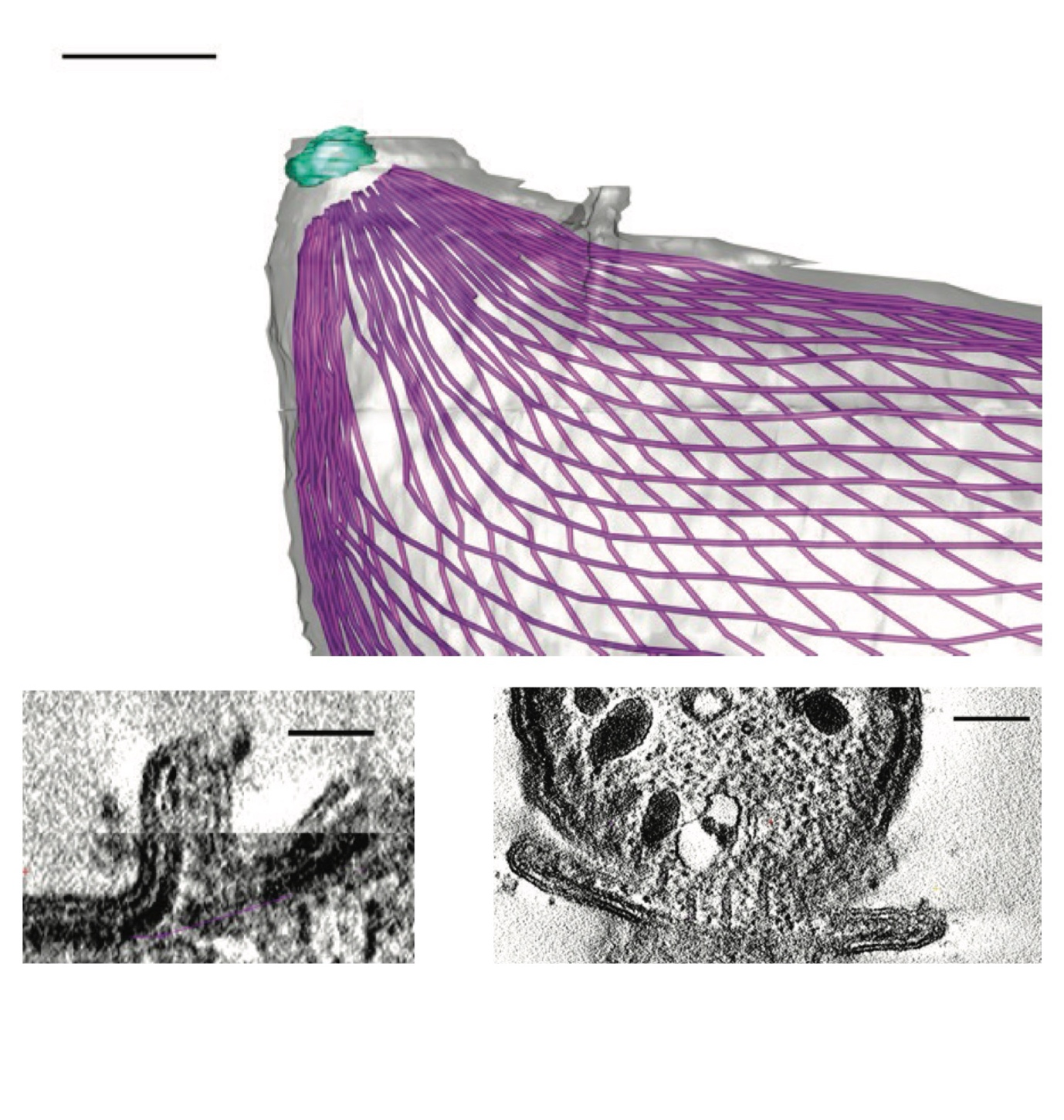


**Supplemental Figure 2: Pellicular folds at the apical end.**

ssET dataset showing the segmentation of conoid (turqouise), microtubules (violet) and plasma membrane (grey) and two single slices from the stack showing that the IMC subtends the plasma membrane but that no microtobules are present in the pellicular folds. Scale bars: Top row: 100 nm. Bottom row: 50 nm


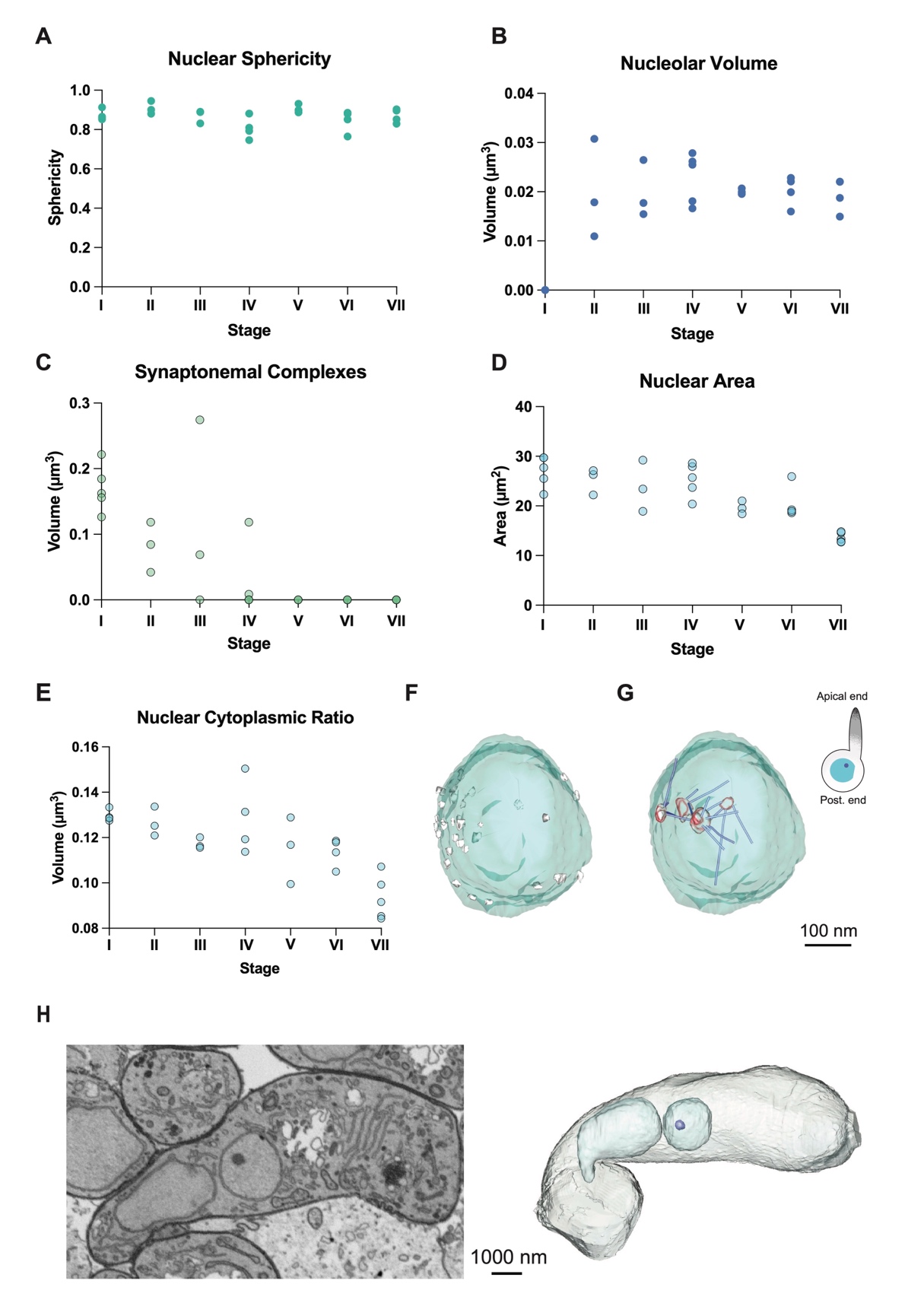


**Supplemental Figure 3: Nuclear features.**
A-E: Nuclear spericity (A), nucleolar volume (B), volume of synaptonemal complexes (C), nuclear area (D) and nuclear cytoplasmic ratio (E).

F-G: Segmentation of the nucleus showing that NPCs (white) are concentrated at the side where centriolar plaques (red; with associated nuclear microtubules, blue) are present. The side of the nucleus facing the apical part of the ookinete remains NPC-free.

H: Nuclear relocalisation observed in FIB-SEM (compare figure 4G). Example shows a nucleus split in two parts.


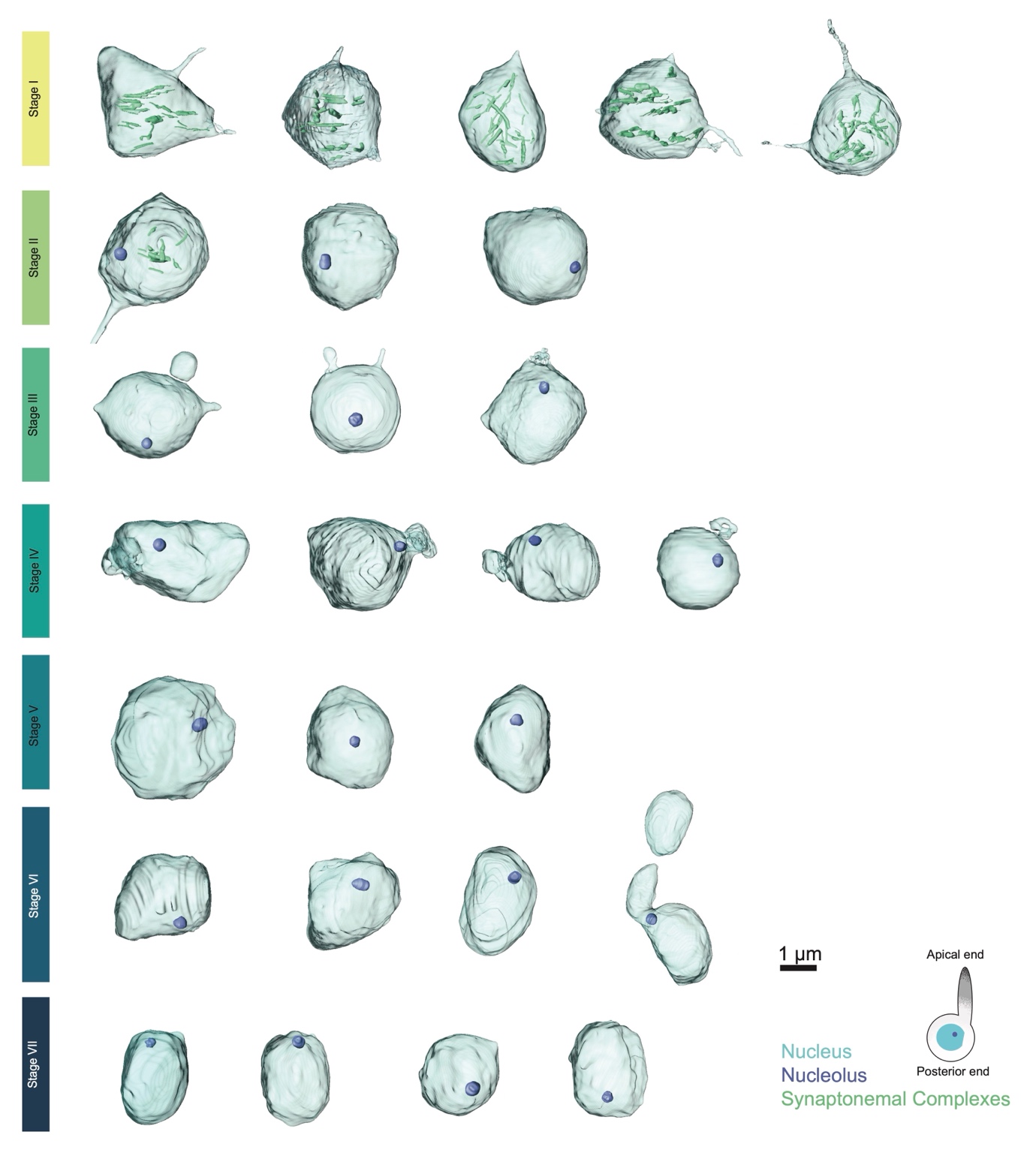


**Supplemental Figure 4: Nuclei.** All nuclear segmentations of each stage oriented along the apical axis of the ookinete. Nucleolus (blue) and synaptonemal complexes (green) are shown. Note the nuclear tails and protrusions in stages I-IV and the split nucleus in stage VI.


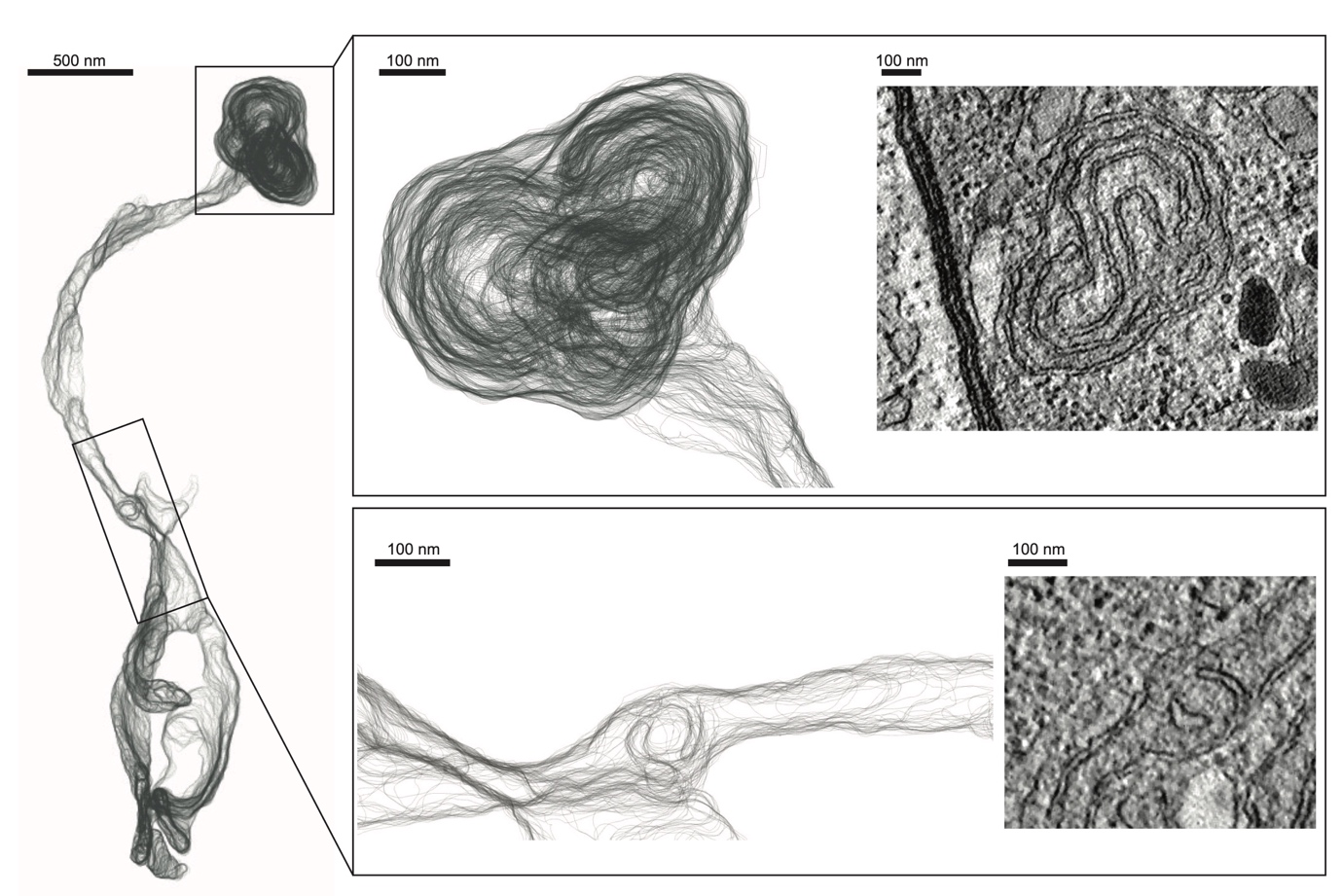


**Supplemental Figure 5: Pleiomorphic mitochondrial structures as oberved from ssET.**

**
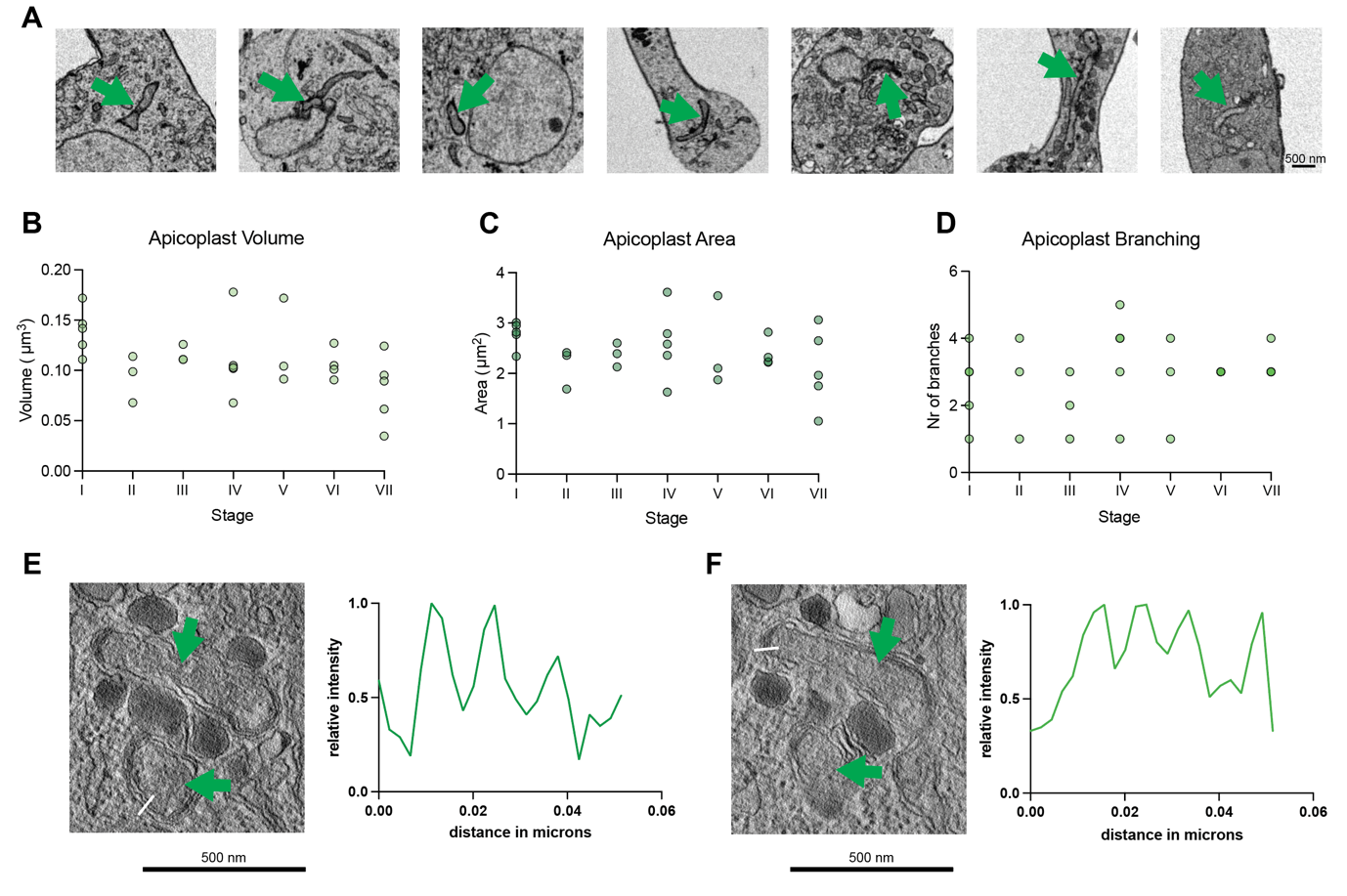
**

**Supplemental Figure 6: Apicoplast morphometrics and ultrastructure**.

A: Single slice from SBF-SEM data showing the apicoplast and its ultrastructural surroundings. Slices cooresprond to Figure 6A.

B-D: Morphometric parameters analysed from SBF-SEM data: apicoplast volume (B), apicoplast area (C) and the branching of the apicoplast (D).
E-F: Single slices from ssET visualizing the apicoplast with intensity plots measured along the white bar showing either 3 (E) or 4 (F) membranes.

**
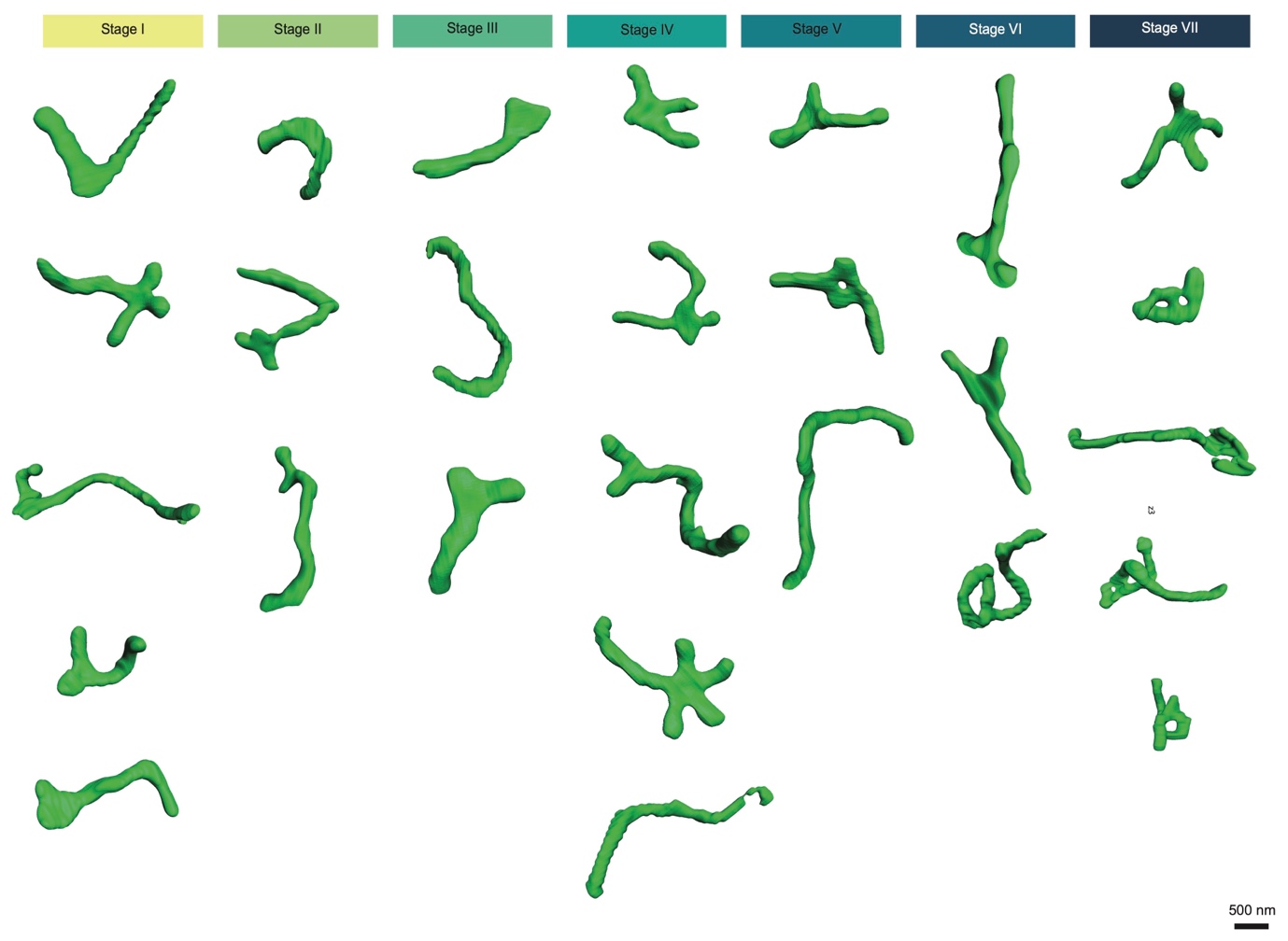
**

**Supplemental Figure 7: Apicoplast morphologies.** All segmented apicoplasts from SBF-SEM datasets positioned to reveal most organellar features across all stages. Scale Bar: 200 nm. No correlation of apicoplast morphology and developmental stage was found.

**
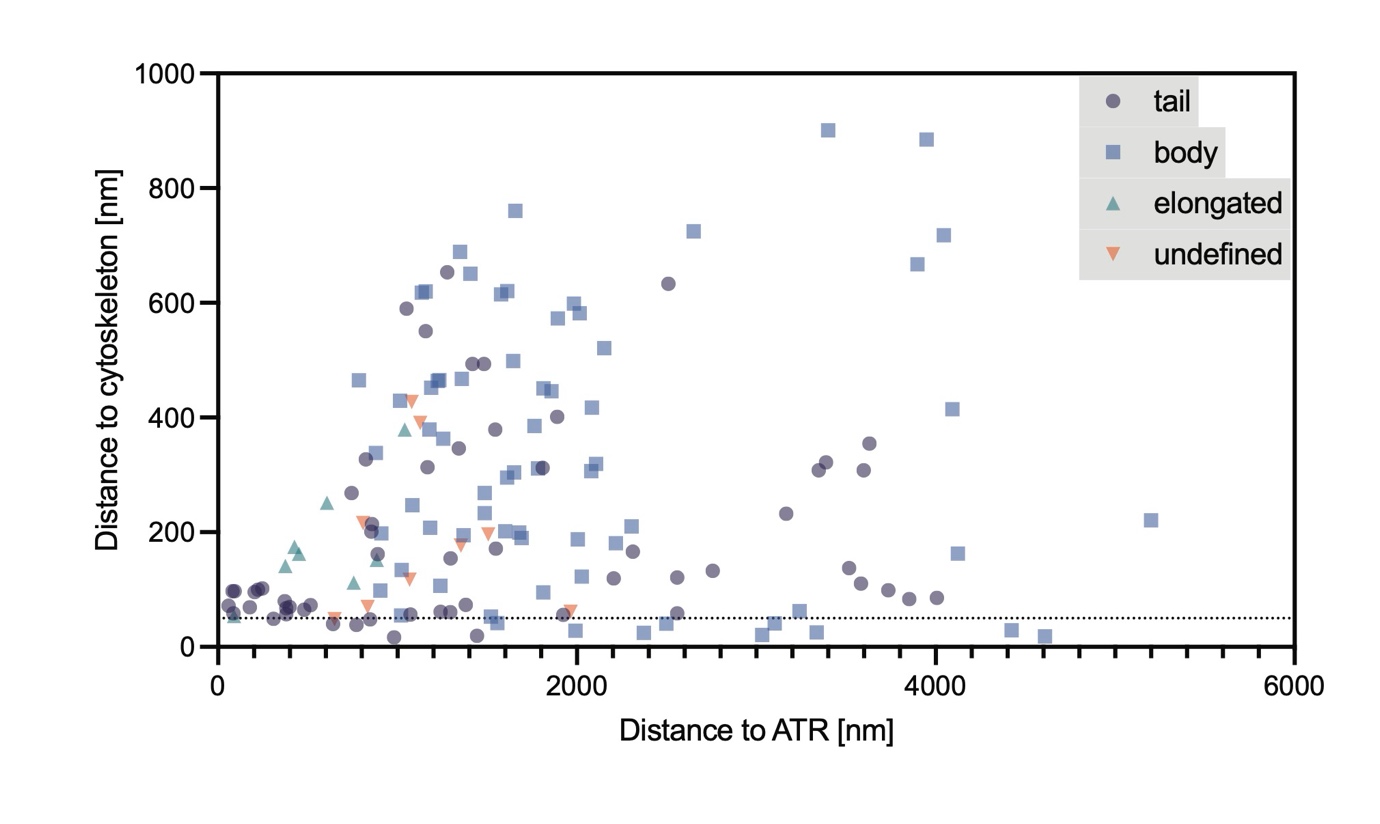
**

**Supplemental Figure 8: Microneme measurements.** Microneme distance to microtubules vs. distance of micronemes to the apical tubulin ring (ATR). Mean distance to microtubules was 260 nm, dotted line indicates 50 nm.

**
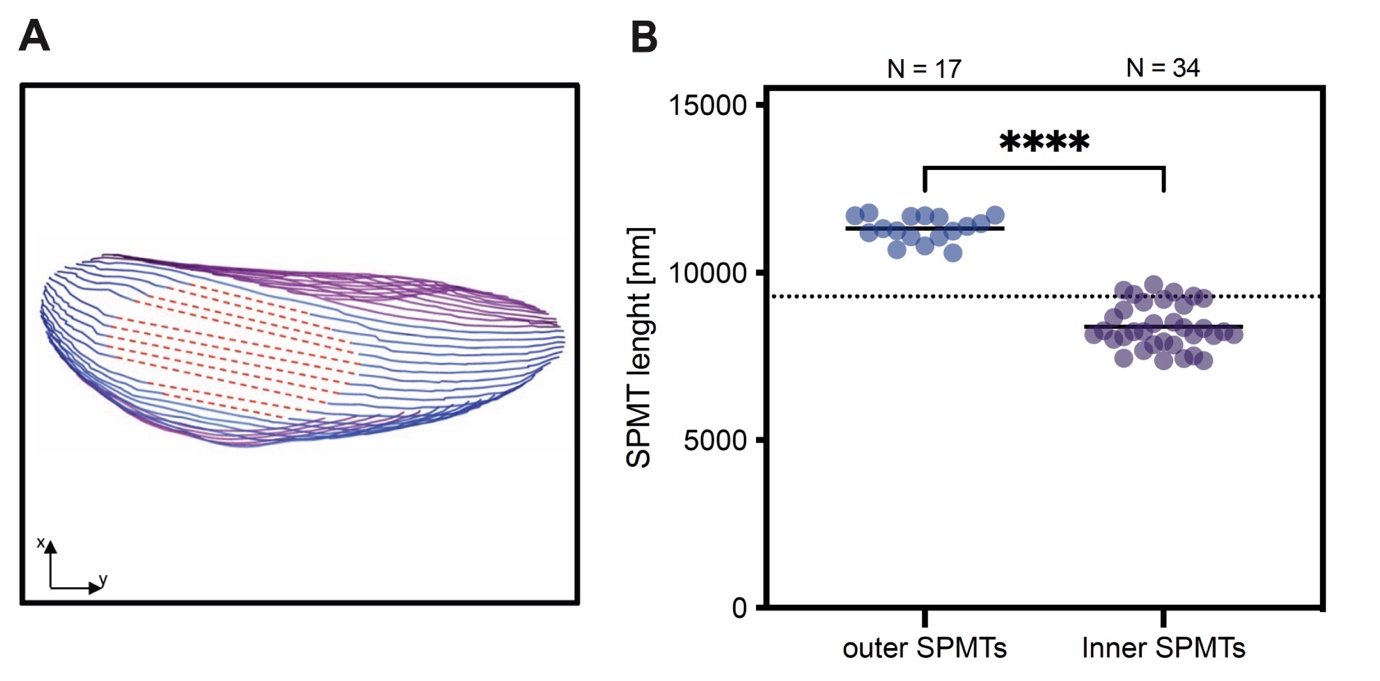
**

**Supplemental Figure 9: Reconstructing microtubules in missing data.**
A: Interpolation of microtubules that could not be fully reconstructed from ssET data, due to missing slices. B: Microtubules at the convex side (blue) are longer than on the concave side (violet) of the ookinete. p < 0.0001 (Mann-Whitney test)
